# Interprofessional Identity in Health and Social Care: Analysis and Synthesis of the Assumptions and Conceptions in the Literature

**DOI:** 10.3390/ijerph192214799

**Published:** 2022-11-10

**Authors:** Gabriël Rafaël Cantaert, Peter Pype, Martin Valcke, Emelien Lauwerier

**Affiliations:** 1 Department of Public Health and Primary Care, Ghent University, 9000 Ghent, Belgium; 2 Research Group Interprofessional Collaboration in Education, Research and Practice (IPC-ERP), Ghent University, 9000 Ghent, Belgium; 3 Department of Educational Studies, Ghent University, 9000 Ghent, Belgium; 4 Department of Experimental-Clinical and Health Psychology, Ghent University, 9000 Ghent, Belgium

**Keywords:** interprofessional identity, interprofessional education, interprofessional collaboration, collaborative practice, healthcare, welfare, curriculum, continuous professional development

## Abstract

Interprofessional identity (IPI) development is considered essential in reducing incongruency and improving interprofessional collaboration. However, noticeable differences in conceptualizations are being put forward in the literature, hindering interpretation of research findings and translation into practice. Therefore, a Concept Analysis and Critical Interpretative Synthesis of empirical research articles were conducted to explore the assumptions and conceptions of IPI. Independent literature screening by two researchers led to the inclusion and extraction of 39 out of 1334 articles. Through critical analysis, higher order themes were constructed and translated to a synthesizing argument and a conceptual framework depicting what constitutes IPI (attributes), the boundary conditions (antecedents) and the outcomes (consequences) of its development. The attributes refer to both IPI’s structural properties and the core beliefs indicative of an interprofessional orientation. The antecedents inform us on the importance of IPI-fitting constructivist learning environments and intergroup leadership in enabling its development. This development may lead to several consequences with regard to professional wellbeing, team effectiveness and the quintuple aim. Given the educational orientation of this study, ways for facilitating and assessing the development of IPI among learners across the professional continuum have been proposed, although empirical research is needed to further validate links and mediating and moderating variables.

## 1. Introduction

Research and practice point at major changes in populations’ needs due to demographic and socio-cultural shifts, warranting a model of care in which medical, psychological and social determinants of health and wellbeing are integrated through interprofessional collaboration [1,2]. Interprofessional collaboration (IPC) occurs when professionals with differing backgrounds share their expertise and work together while recognizing professional differences and acknowledging interdependence [3].

However, IPC can be hampered due to distinct professional identities in which the set of values, norms, traditions and beliefs shared within a professional community are internalized by the professional [4,5]. An individual’s professional identity is important as it fosters the “ongoing process of interpretation and reinterpretation of experience” [6] (p. 9). Nonetheless, the self-categorization within a certain professional group might also lead to an overemphasis on similarities (‘We’ instead of ‘I’) between group members, resulting in the avoidance of identity-incongruent interactions (‘We’ versus ‘Them’) with other professionals [7]. The downside of this categorization is that misconceptions and unfamiliarity with other experts’ roles, goals and perspectives may lead to stereotyping, which is characterized by the firm belief in the value of one’s own ideas and practices while devaluing those of others [8,9]. Hence, a strong emphasis on one’s professional identity may lead to poor collaboration and a bad fit with the requirements of the specific care organization [10,11]. 

Education, starting from early informal life experiences and enhanced by formal classroom experiences and workplace learning, contributes to professional identity development, in which the nature and organization of educational activities defines a student’s identity attributes and influences the process of categorization [12,13]. This is particularly obvious when looking at the organization of higher education locked into academic silos, potentially leading to students developing a distinct (uni)professional identity during their training in becoming, for instance, a physician, a nurse, or a social worker [4,5]. InterProfessional Education (IPE), in which students are involved in a co-learning process and are encouraged to build meaningful relations with students from other disciplines, could help in countering the risk of unfavorable early categorization. This IPE entails developing—building on pre-existing—beliefs pertaining to the value and importance of collaboration as well as gaining the skills to act upon these beliefs [5,13]. This could help developing an interprofessional identity (IPI) from which durable interprofessional behavior may ensue [13,14]. 

Notwithstanding the increasing research interest since the 2000s, no standard or shared conceptualization of IPI is available, hindering theory construction and translation into practice [12,15]. A comprehensive conceptualization could be achieved through identification, analysis and synthesis of the existing assumptions and conceptions of IPI in the literature followed by a critique and theoretical analysis [16]. This brings us to the central research question of the present paper: ‘What are the assumptions and conceptions of IPI in the literature?’. This question is studied by focusing on two sub-questions: (a) What theoretical frameworks have been adopted in the literature to interpret IPI? and (b) What are the attributes, antecedents and consequences of IPI in the literature? 

## 2. Method 

### 2.1. Philosophical Perspective

The ontological perspective adopted in the present study aligns with relativism and subjective idealism, introducing an emphasis on multiple mental constructions of the same reality by different individuals [17]. It also implies that the meaning of concepts is not fixed, might evolve over time, is contextually bound, and is driven by multiple perspectives based on a variety of conceptions [18]. This perspective influences the adoption of an inquiry corresponding to a constructionist epistemological view: knowledge is socially constructed through cognitive activities, such as interpretation, to generate contextual understanding and make sense of reality [17,18]. In operational terms, we developed a knowledge base by analyzing and synthesizing (re)interpretations from primary research, into a meta-construction [19]. This approach to theory building is highly interpretive and to guarantee a sufficient level of synthesis, both a critical interpretative synthesis (CIS) and concept analysis (CA) methods were combined to generate novel interpretations transcending primary study findings while also taking the context into account [17,20]. Given the educational orientation of this paper, our philosophical stance also fits state-of-the-art approaches to workplace learning and adult education that introduces a corresponding lens while looking at the available literature. For instance, Beckett [21] (p. 41) emphasizes the personal (re)interpretation of experiences before and during workplace activities when “people bring to work their entire experiential selves”. This fits adult learning approaches as reiterated by Andresen et al. [22] and Arghode et al. [23], who assert that learning is an active and holistic construction process derived from socially constructed and contextualized experiences, which promotes a better understanding of the self. Accordingly, the CIS and CA are expected to contribute to clarifying how formal and informal educational opportunities are related to IPI. 

### 2.2. Research Design 

In view of the CA, we adopted the model of Walker and Avant [24] that consists of eight steps, following three iterative stages. In the first stage, the assumptions and conceptions pertaining to the attributes of IPI were obtained and analyzed through careful inspection of the literature. These attributes refer to frequently reappearing perspectives, characteristics, elements, or properties associated with IPI. In the second stage, the antecedents and consequences were identified and synthesized. The antecedents are the incidents and conditions that precede the occurrence of the concept, while the consequences refer to the resulting events, actions and outcomes. At the end of the process, vignettes (Table S1) were created to help contextualizing IPI and to offer a sound theoretical base to ground educational approaches. In these vignettes, short hypothetical narratives of interprofessional scenarios are portrayed to facilitate readers in gaining a practical understanding and helping them discern the degree of learners’ IPI development [24,25]. In the third stage, empirical referents in terms of the measurable aspects of IPI were discussed to allow assessment of the presence or change in the concept. During the first and second stages, we also adopted the CIS approach of Dixon-Woods et al. [16] to review the literature in search of the attributes as well as synthesize the antecedents, attributes and consequences. The PRISMA-statement for conducting systematic reviews and the ENTREQ reporting guideline for the synthesis of qualitative research were consulted to warrant transparent reporting of both the research process and the results [26,27]. The checklists of both have been added to the supplementary files (Table S2). 

### 2.3. Search Strategy

The search strategy followed an iterative approach in view of achieving theoretical saturation [16]. A first literature search comprised consulting the evidence base to gain a general understanding with regard to the theories, trends, commonly used methods and research gaps pertaining to IPI [20]. This search helped in starting a second literature search with a search string combining the keywords interprofessional and multidisciplinary, transdisciplinary, cross-disciplinary, interdisciplinary, collaborative, team, intergroup and dual, each in combination with ‘identity’. No Medical Subject Headings (MeSh) terms or other relevant headings could be identified. PubMed, Embase, Web of Science, Scopus, ERIC, Cinahl and PsycArticles were searched in February 2020, leading to the identification of 1.313 records. The subsequent consultation of Google Scholar, inspection of relevant journals such as Journal of Interprofessional Care and Medical Education, and screening of reference lists and citations resulted in 16 additional records. A third literature search was set up in July 2020, with a search string (Table S3) enriched with terms that emerged during earlier steps: reconfigured professional identity, care unit identity, superordinate identity, common identity, crossed identity, interprofessional role identity, extended professional identity, group identity, social identity and collective identity. This search yielded 32.192 records. 

### 2.4. Study Selection

To allow for a critical synthesis of the research, both quantitative and qualitative empirical studies as well as other types of adjunct literature such as theoretical works, editorials, commentaries, grey literature, program evaluations and case studies were considered eligible [16,19]. This implied the inclusion of all—English language—literature in which IPI (or related concepts) was explicitly mentioned along with sufficient context to be considered theoretically relevant for interpretation. Accordingly, brief mention of the concept, for instance in abstracts and posters, was an argument for exclusion. 

**Figure 1:**
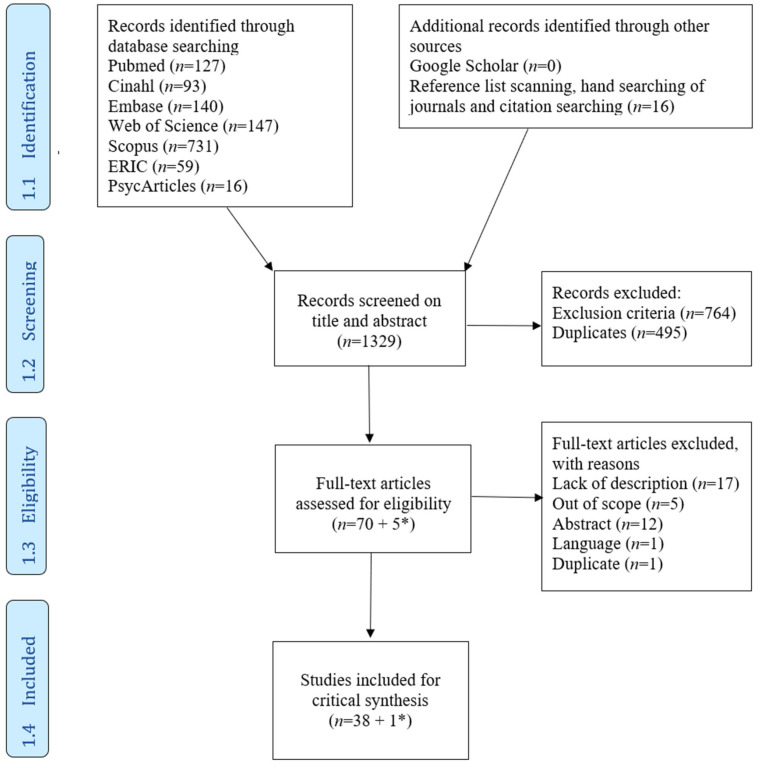
PRISMA flowchart of included studies after the second and third literature search (* represents the articles that were added after the third literature search).

The Rayyan online screening tool was used to import the records identified during the second literature search and allow blind screening of the title and abstract [28]. After removal of duplicates (*n* = 495), the screening was carried out independently by the first and last author, which led to the exclusion of 764 records not meeting the inclusion criteria. Rating conflicts (*n* = 29) pertained to articles that either mentioned an identity of organizations in a collaborative setting or discussed a scale to measure team identity. It was decided to retain these articles. The percentage agreement (96.5%; >75% recommended) and Cohen’s Kappa (0.64; >0.61 recommended) were calculated with the ReCal online tool to determine inter-rater agreement in relation to the screening and selection of outcomes [29,30]. Next, 70 out of the 1.329 articles eligible for full-text screening were imported in Endnote X9 citation manager and screened independently by both authors. This screening led to the inclusion of 38 out of 70 articles with a high inter-rater agreement of 83% and a Kappa of 0.65. Rater conflicts (*n* = 13) mostly resulted from ambiguities in concepts or unclear reporting in the articles but were resolved after discussion. As all benchmarks were met, the third literature search and screening of 32.192 records on the base of the title and abstract were further continued by the first author. This screening resulted in five additional articles eligible for independent full-text screening by both authors, resulting in one extra article meeting inclusion criteria. In total, 39 articles were included for analysis and 36 out of 75 were excluded (see Table S4 for excluded studies [31,32,33,34,35,36,37,38,39,40,41,42,43,44,45,46,47,48,49,50,51,52,53,54,55,56,57,58,59,60,61,62,63,64] with related rationale). The PRISMA flowchart (Figure 1) represents the results of the second literature search with the results from the third literature search, which have been added by means of an asterisk. 

### 2.5. Data Extraction and Analysis

A standardized data-extraction form was created to position the literature within its context, to provide an overview of frequently used concepts, and to facilitate the identification of relevant themes [20]. Extraction focused on: (a) author, country and title, (b) aim of the study, (c) research design and data collection, (d) theoretical perspective and conceptual base, (e) antecedents, (f) attributes, and (g) consequences. In each case, the entire full text was extracted instead of focusing on specific sections. Articles were sorted by the concepts being described and same-author studies were grouped in the data-extraction table (Table S5). The majority of included articles (*n* = 25) explicitly mention IPI, most often in combination with the concept dual identity (*n* = 11) [65,66,67,68,69,70,71,72,73,74,75,76,77,78], but also in combination with roles (*n* = 4) such as: change agent [79], team player [80,81], boundary spanner [82,83] and interprofessional [84]. Furthermore, IPI was described as a flexible (*n* = 1) [85], integrated (*n* = 1) [86] or an extended (*n* = 2) [87,88] professional identity. Additionally, team identity (*n* = 8) was described as a standalone concept in the remainder of the articles focusing on IPC and IPE [65,66,89,90,91,92,93,94]. Other relevant identified concepts were: collective identity (*n* = 2) [95,96,97], superordinate identity (*n* = 2) [98,99], health care professional identity (*n* = 1) [100], group identity (*n* = 1) [101], and collaborative identity (*n* = 1) [82,102]. The most widely adopted theoretical perspective (*n* = 25) was Social Identity Theory (SIT), which approaches IPI as a dual role and/or a flexible/integrated/extended professional identity [65,66,68,69,70,72,73,74,76,77,78,80,81,84,85,86,87,88,92,94,98,99,100,103]. A theoretical perspective on identity was absent in six articles [67,89,91,93,95,101], while other articles made reference to social learning theories, such as Communities of Practice (*n* = 3) [82,96,97,102], Role Identity Theory (*n* = 1) [80,81], Organizational Learning Theory (*n* = 1, e.g., double-loop learning) [90], discourse and narratives (*n* = 2) [75,96,97] and Bhabha’s concepts of hybridity and third space (*n* = 1) [71]. 

In line with the CIS methodology, an iterative and inductive analysis approach was adopted to create, define and apply codes [19]. The unit of analysis for generating a code corresponded to a section reflecting an antecedent, attribute and/or consequence that was used to organize the data-extraction form. More specifically, text passages were coded systematically into descriptive and analytical themes, independently by the first and last author. A coding book, comprising definitions, memos and examples of codes and themes (Table S6) was developed and pilot tested after independently analyzing ten identical articles to determine and resolve potential discrepancies and bias in interpretations. The remainder of the articles were analyzed with the revised coding book by the first and last author, allowing for a more transparent and reliable data analysis approach. In view of reflexivity and researcher triangulation, each author reflected upon their specific expertise and perspective pertaining to IPI and recurrent discussions were held to accommodate multiple perspectives, tackle ambiguities and discuss memos [16,19]. 

On the base of the codes presented in the data-extraction table (Table S5), a total of nine attributes, six antecedents and three consequences were synthesized (Table 1) through abstraction, integration and generalization.

Without repeating what is specified in the table, we emphasize how the attributes reiterate the first conceptual orientation towards IPI as discussed earlier in the article. The list of attributes also illustrate the holistic impact of IPI on individuals as a person and professional: five mirror content-related features and four elucidate structural properties. The content-related features comprise the core Values (*n* = 20) that professionals hold (e.g., respect), their Awareness (*n* = 8) of preconceived assumptions (e.g., valuing of collaboration) as well as the Openness (*n* = 10) to professional diversity (e.g., being positive towards shared goal setting), the perceived Self-Efficacy (*n* = 28) in their capabilities (e.g., being confident to share leadership) and their Commitment (*n* = 24) to an interprofessional group or community (e.g., perceiving a sense of belonging). Next, the first two structural properties emphasize a Context-Dependency (*n* = 11), which implies the fit between the professional and the interprofessional environment as well as highlights the development of a Team Mental Model (*n* = 8), inferring a congruence between team members (e.g., sense of working side by side). The last two structural attributes depict the development of IPI as Fluid and Dynamic (*n* = 15) throughout the lifespan of a professional (e.g., through negotiated experiences) and by means of Calibration (*n* = 9), through which the cognitive, social and emotional capacities required for the meaning-making of collaborative practice are integrated (e.g., transcending professional boundaries). The list of antecedents refers to the nature of the contexts in which learning occurs, such as through Curricula (*n* = 8, e.g., focusing on continuing education), use of Educational Strategies (*n* = 21, e.g., e-portfolio) and the emphasis on explicit Role Learning (*n* = 15, e.g., building knowledge about other disciplines). The list also points at conditions and contextual characteristics that enable IPI, such as Breaking Down Barriers (*n* = 17, e.g., challenging misconceptions) that hinder interprofessional reflection, the adoption of an Intergroup Leadership approach (*n* = 7, e.g., participative decision-making) and the investment in a fertile Community of Practice (*n* = 9, e.g., frequent informal encounters). IPI has also been found to result in a series of consequences that reflect a multi-level perspective on what IPI implies and impacts in terms of individuals’ Professional Wellbeing (*n* = 12, e.g., feeling empowered), Team Effectiveness (*n* = 12, e.g., improved team communication), and Health System Performance (*n* = 7, e.g., higher quality of care). 

Throughout the subsequent analysis phase, these descriptive and analytical themes were critiqued by identifying flaws or contradictions in research traditions and metanarratives and were evaluated in view of their frequency of occurrence and theoretical relevance through constant comparison to generate higher order themes [16,20]. Ultimately, a critical synthesis of the higher order themes was performed at conceptual and theoretical levels to translate the novel interpretations into a synthesizing argument and construct a conceptual framework, which is reported in the Results and Discussion section [16]. 

## 3. Results and Discussion

### 3.1. Summary of Results

The aim of the present study was to develop a synthesizing argument and conceptual framework pertaining to interprofessional identity, based on a concept analysis and critical interpretative synthesis of the assumptions and conceptions of IPI in the literature. Analysis of 39 identified articles resulted in the delineation of IPI-related attributes, antecedents and consequences that have been synthesized by means of critical and theoretical analysis in terms of how the factors could be interlinked, thereby explaining how and why IPI develops in an individual and what its impact is. As an illustration, several scenarios of how the development of this identity may present itself have been provided as vignettes in the supplementary files (Table S1). This critical synthesis further helps with connecting our findings to the available IPI literature, but also allows us to project ideas about ways to develop IPI in future professionals and how to measure its components and the outcomes. 

**Figure 2:**
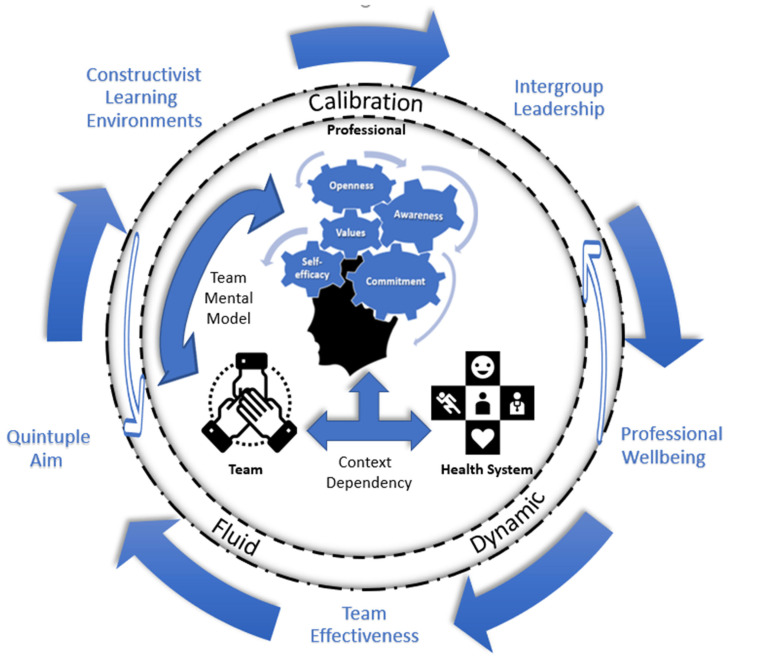
Conceptual framework visualizing the interconnectedness of the antecedents, attributes and consequences of IPI. The inner circle shows the five content-related attributes that need to be integrated into a professional’s identity as well as illustrates the reciprocal influence between professionals through a team mental model, while emphasizing the context-dependency of the former in the health system. The middle ring argues that professionals, teams and eventually the health system continuously develops in a fluid and dynamic way and through calibration. The outer region encompasses the antecedents (**top part**) which indirectly leads to the consequences (**bottom part**) while both also influence and are influenced by the IPI development depicted inside the circle and inferred by the dotted lines.

The present Results and Discussion section constitutes the synthesizing argument, starting with a graphical representation by means of the established conceptual framework (Figure 2). This figure depicts the synthesized higher order themes resulting from the critical theoretical analysis of the attributes, antecedents and consequences as well as shows their interrelatedness. Accordingly, we explore these higher order themes step-by-step in the next paragraphs to provide an integrated discussion of the complexity of IPI, where we go beyond a simple descriptive approach of the isolated themes shown in Table 1. We start with elaborating on the content-related features of IPI—central in the figure—comprising the values, awareness, openness, self-efficacy and commitment at the level of a professional. Afterwards, we discuss the structural properties—within the inner circle—by emphasizing the context-dependency of IPI in relation to the health system and by focusing on the congruency within a team through the development of a team mental model (TMM). We then comment on the continuous development of IPI in a fluid and dynamic way and through calibration, which is inferred by the inner ring. The antecedents—above the circle—further help us to operationalize adequate IPI-fitting constructivist learning environments (CLE) based on collaborative, constructive and contextualized learning principles. Additionally, they inform us on the importance of intergroup leadership in addressing conditions and contextual characteristics that enable IPI development—hence the permeable inner lines—through educational redesign and by constructing an interprofessional CoP. Ultimately, these antecedents may lead to several consequences—shown in the lower half—with regard to professional wellbeing, team effectiveness and the quintuple aim, which will be explored accordingly. In turn, these consequences are believed to impact the antecedents as well as exert influence on the development of IPIs, again emphasized by the permeable barriers. This synthesizing argument will then be followed by an examination of the measurability of IPI by means of defining the empirical referents to inform us of effective assessment practices. Lastly, we consider the strengths and limitations inherent to this research article before ending with our conclusions. 

### 3.2. Attributes

#### 3.2.1. Content of IPI

Interprofessional identity is a multidimensional construct, which is apparent when looking at the five content-related attributes. These attributes refer to the knowledge structures within the cognitive system that were synthesized as follows: values, awareness, openness, self-efficacy and commitment. While being deeply rooted in one’s identity, these attributes guide the perceptions, interpretation, judgements and overall meaning-making of individuals towards an interprofessional instead of a professional-centric way of thinking, feeling and acting [104,105]. These attributes will be discussed further in respective order. 

Values. The literature synthesis activity resulted in six co-dependent values associated with an interprofessional orientation. They represent basic convictions that guide professionals to enact collaborative behavior across interprofessional situations. The first value that professionals ought to mirror is a moral responsibility of collaboration, following which they feel responsible to provide patient-centered collaborative care that is responsive to patients’ needs, values and preferences [106,107]. Associated with this responsibility is the shift towards interdependence of professional practice, emphasizing the convictions of not being able to provide patient-centered care in a completely independent way [108]. A prerequisite for this interdependence is the value of equality, in which professionals have an innate desire to share decisions and recognize each other’s unique contributions [108,109]. This equality infers a constructive use of power, meaning that influence is exercised on the basis of relevant expertise, competence and information as opposed to a destructive use based on authority [110]. Furthermore, professionals may also hold a desire to feel involved and liked in the institution by other professional groups, characterized by a feeling of togetherness [94,111]. The respect for the competence and the complementarity of other professionals’ expertise is emphasized with the fifth value, which also is considered a precursor for developing trust between professionals. These last two values are also believed to be indispensable in establishing durable interprofessional relations and are characterized by the mutual acceptance and tolerance of differences in values, beliefs and behavior and the willingness to share their expertise, opinions, thoughts and feelings [94,112,113]. 

Awareness. This second content-related IPI attribute points at an awareness about beliefs and stereotypes that might hinder collaboration. This awareness is implied, such as those regarding the expectations and convictions about the value of collaboration as well as the perceived similarities and differences between professionals. By being aware and able to challenge and alter these assumptions, a greater acceptance of the differences may result due to a more congruent belief system that connects rather than separates professional practices [92,105,114,115]. 

Openness. The third content-related IPI attribute means that professionals are not bound by their traditional boundaries of profession-centric thinking, but instead have developed diversity beliefs that counter unfavorable categorization and allow a broader orientation on client-centered collaborative care [116,117]. This orientation is associated with the valuing of a diversity of other professionals’ perspectives and the willingness for joint action through active involvement [92,117]. Openness could also imply developing a sense of confidence in one’s professional role and the ability to share roles as well as possessing the required knowledge and beliefs, leading to a greater willingness and readiness for participating in interprofessional education and practice [86,117,118]. 

Self-efficacy. This fourth content-related attribute is closely linked with the previous one as self-efficacy refers to beliefs “concerned with judgments of how well one can execute courses of action required to deal with prospective situations”, including those in interprofessional situations [119] (p. 122). As evidenced in this study, several interprofessional capability clusters were identified pertaining to the knowledge and abilities about professionals’ roles and responsibilities, leadership, teamwork and communication. Whilst these capabilities were associated with IPI in the majority of articles, some studies assert that being competent and ‘knowing how’ to do something does not necessarily result in having a sense of efficacy to collaborate or vice versa. Self-efficacy primarily entails what someone ‘believes he is capable of’ doing, which also is dependent on one’s physical and mental state as well as the context [120,121]. When professionals perceive a high self-efficacy, their motivation, performance and wellbeing may increase due to increased effort and greater persistence [121,122]. Hence, self-efficacy is essential within an IPI as the related beliefs may similarly determine the engagement of professionals and what they actually are capable of doing when feeling comfortable and confident. 

Commitment. The fifth content-related attribute refers to the self-reflective beliefs about someone’s relationship with social groups or adoption of roles [123]. The majority of related studies focus on the commitment in relation to groups through the lens of Social Identity Theory (SIT), while a minority utilized Role Identity Theory (RIT) to explain one’s commitment in relation to roles. According to the unified identity theory from Stets and Burke [124], both theories can be combined to define these types considering their impact on enacting the values, beliefs and norms associated with such groups and roles [125]. We first elaborate on the former before discussing the latter.

- Groups. Being committed to occupational or organizational groups, and in this case an interprofessional community, is beneficial as it is an “essential human desire to expand the self-concept to include connections with others and to feel a sense of belonging” that makes members more willing to contribute to group efforts [10] (p. 334), [125]. This view on commitment and sense of belonging has been commonly adopted in the current IPI literature, especially since the development of the Interprofessional Socialization Framework by Khalili et al. [126]. This framework is based on the emerging concept of dual identity, a type of social identity, in which it is believed that professionals can develop at the same time a commitment to both their own professional group and to an overarching group of interprofessionals [126]. Dual identity closely resembles the collective identity perspective adopted by Crepeau [95], Thistlethwaite et al. [96] and Hean and Dickinson [98], following which professionals also commit to an overarching group in pursuit of a common goal. However, both collective and dual identities may be less suitable for the health and social care domain for two reasons: (a) in case of a collective identity, professionals have to relinquish their group distinctiveness that leads to a blurring of role boundaries, and (b) a dual identity requires a strong commitment with the subordinate professional as well as the superordinate interprofessional group [9,127]. Attempting to establish an identity in which group distinctiveness needs to be discarded is bound to fail considering that professionals in health and social care tend to develop a strong commitment and a desire to maintain their unique identity [127]. Similarly, developing a dual identity proves challenging when a strong interprofessional commitment implies subsumption of a professional commitment [128]. This implies that different professionals’ groups should become balanced in terms of hierarchy and power, which is difficult to achieve when distinct professions feel they might lose their dominant position, and hence their uniqueness, in relation to other groups. Additionally, the accountability for professional actions is in our health care system described in line with distinctive professional duties, which automatically reinforces power differences [65,127,129]. For this reason, promoting a dual or collective identity could actually hinder the development of an IPI, due to a higher resistance from ‘rigid’ uniprofessional identities that may induce identity threat, conflict and distrust in leadership [9,127,129].

Evidently, a more suitable alternative type of identity is needed that ensures compatibility of professional commitments. Reinders et al. [130] emphasize the importance of this compatibility and posit with their extended professional identity theory that professional and IPI are two distinct social identities, of which the former is a subordinate and the latter is a superordinate social identity. Accordingly, professionals could simultaneously develop a strong commitment to their own professional group as well as to a ‘wider circle of group membership’, as expected in an interprofessional group [87]. Alternatively, promoting an Intergroup Relational Identity (IRI) may be relevant considering the emphasis it puts on intergroup relations built on mutual values, goals and a shared vision, while maintaining the distinctiveness between professional groups [9,127]. According to Rast et al. [128] (p. 1) ”an intergroup relational identity refers to a form of social identity that is defined in terms of the cooperative and mutually promotive relationship between subgroups”. With the IRI, professionals appear to develop an interprofessional commitment through the internalization of intergroup relations, in which they identify with a shared vision, acknowledge their differences in terms of strengths and weaknesses and value respect and collaboration [9,131]. In sum, the difference with dual, collective and extended professional identity could be that the sense of belonging to an interprofessional group develops through a commitment to collaborative relationships instead of a commitment to a collective or to both the professional and interprofessional group [128]. Note that social identities may infer different foci such as in terms of occupational, organizational or team commitments that may differ and even be in conflict [125]. Although we found a somewhat clear distinction between occupational and team identity in the reviewed literature, the fit between organizational identity and IPI remained unclear and requires further inquiry.

- Roles. The commitment in relation to roles follows a different perspective as it rather focusses on the intrapersonal level and on the meaning individuals associate with the roles they occupy in society [124]. Roles are socially constructed in terms of expected activities, rights, obligations, beliefs, behaviors and boundaries and a combination of these roles may constitute a profession. Traditionally, professionals in health and social care primarily assume the role as an expert and a professional. However, changes in society warranted the creation of new roles in light of the emerging challenges that require a collaborative approach, such as those reflected in the role as a team player, boundary spanner, interprofessional and change agent [79,80]. The concept of a T-shaped professional is especially relevant here, following which professionals are regarded as collaborative innovators on the basis of their roles as boundary spanners and team players with which they are able to facilitate collaboration beyond functional boundaries [132,133]. This evolution in roles is also apparent in the CanMEDS competency framework. In this framework, the key competencies of ‘the effective and responsible physician’ were translated into seven roles that have been adopted by various other professions [134,135]. Accordingly, professionals adopt the central role of the expert that mirrors their professional field and six intrinsic roles: communicator, collaborator, leader, health advocate, scholar and professional [135]. The centrality of the expert role could correspond with what Kislov et al. [82] define as a stable ‘core identity’ that is surrounded by an ‘extended identity’ that is subject to change, depending on what is experienced and learned over time. With this in mind, one could theorize that a commitment to the role of collaborator, or other closely related roles, could be inherent to developing an IPI. The leadership role is one such related role, which can be associated with developing a leader’s identity. This identity has gained considerable attention in the literature due to the growing societal expectations that all professionals should learn to engage in shared or collaborative leadership [136]. These leadership styles are characterized by being comfortable and passionate in working interdependently towards group or organizational goals through the active involvement of each other’s perspective. This way, power and responsibility become shared on the basis of situational expertise rather than being concentrated in one discipline [136,137]. Correspondingly, professionals can adopt multiple professional identities that become activated in a suiting context, which further leads us to discuss the structure of IPI.

#### 3.2.2. Structure of IPI

The structural properties of an identity relate, among other things, to its cohesiveness, coherence, saliency, stability, and sharpness of boundaries [5,138]. These properties have been captured in two attributes that define the context-dependency and describe the notions of the TMM associated with IPI. The two other attributes specify the developmental trajectory in being fluid and dynamic as well as explain the nature of calibration in relation to IPI development. However, before elaborating on each of the four attributes, we emphasize the importance of congruency in the different content-related attributes as individuals desire a sense of consistency and security in their thoughts, feelings and behaviors [139,140]. This congruency has been insufficiently delineated in CanMEDS and other competency frameworks. Additionally, the majority of reviewed studies mainly focused on a single or only a few attributes, thereby overlooking the complexity of IPI. The present critical synthesis resulted in a more comprehensive IPI picture that at the same time puts forward the need for congruency when integrating the attributes into a professional’s sense of self through the alignment of cognitive structures. In this way, a ‘coherent identity’ can be developed that helps professionals to accept their complex belief systems and makes them feel confident in acting accordingly. This implies that future research should adopt a more comprehensive take on IPI. However, it also implies that educational initiatives have to consider the complex interrelationship between the different content-related attributes during the cognitive and socioemotional development of learners. 

Context-dependency. Dependency or congruency with the context infers a fit between the individual and an interprofessional environment, which is an important determinant in the salience of IPI. This salience refers to the probability of an identity to become activated on the basis of (sub)consciously perceived contextual cues, which stimulates the enactment of a given identity [124,125]. Accordingly, contextual cues within interprofessional environments, settings or work encounters trigger the IPI. A typical example is the intensive care unit, where mutual reinforcing actions from different disciplines, such as medical specialists, nurses, and laboratory assistants are required to respond to cues related to, for instance, acute and life-threatening situations. Conversely, situations without such cues may not lead to an activation of IPI, which might be the case during regular consultation between a professional and a client [124]. Hence, the learning and possible un-learning of certain associations impact IPC, making it essential to account for this salience in continuous professional development. The latter introduces an interesting avenue for further research. 

Team Mental Model. This second structural attribute can be regarded as a shared mental model of collaborative teamwork, which builds on a mutual commitment of professionals working in an interprofessional team. More specifically, TMM refers to the mental representations that team members share and use to describe, explain and predict group-level phenomena. It makes them feel part of a team where everyone works side-by-side and seems to be on the same page [141]. In this case, the TMM consists of a mutual awareness, shared understanding, and joint agreement of the interprofessional team’s structure, interactions and tasks execution, leading to a proactive, coordinated and mutually predictable exchange of information [141,142]. Both social and cognitive psychology, with the literature mainly pertaining to team identity, helped to explain the impact of this attribute by focusing on the professional diversity within teams [143]. A closely related yet distinct theoretical explanation builds on the transactive memory system (TMS) described in Lave and Wenger’s Communities of Practice (CoP), a specific social learning theory [142]. A CoP represents a persistent and sustained social network characterized by a mutual engagement, joint enterprise, and a shared repertoire of individuals who share a knowledge base through a TMS [5]. TMS refers to the collective awareness and understanding of ‘who knows what’ in terms of overlapping knowledge and domains of expertise, which allows for a coordinated use of the knowledge stored within the community [5,144]. Although the notions of both TMM and TMS are relatively new in the health and social care literature, both seem promising concepts to adopt in future educational studies as they offer a theoretical understanding of interprofessional group dynamics on team performance, and in particular the impact of IPIs in (in)congruent teams [144]. 

Fluid and dynamic. The third structural attribute refers to the ongoing process through which one integrates their past, present and future self, which proceeds in a nonlinear way following steady and turbulent stages [125,145,146]. Such turbulence results from what Jarvis-Selinger et al. [147] label as ‘emerging crises’ that arise when individuals experience dilemmas or critical incidents that challenge their identity, prompt a reevaluation of the situation and eventually lead to a better understanding of the world and personal identity. Within the domain of lifespan and developmental psychology, authors help describing these stages; for instance, Erikson [148]. Central to his theory is the idea of introjection, or the process through which a person adopts certain values, ideas and preferences through socialization or encounters with others. Successful integration relates to the process through which a person has managed to assimilate different identifications into one integrated ‘sense of self’ [149]. The dynamic nature of IPI is also reflected in its non-sequential development as individuals are often confronted with periods of regression when progressing through subsequent identity stages [5]. These stages refer to the cycles of exploration-commitment, during which possible alternative identities are considered and commitments are evaluated under the influence of both intergroup and intra-individual processes such as affect and motivation [148,149]. This structural attribute is important when discussing IPI education: reflective learning should be supported when dealing with emerging crises and introjection and integration should be facilitated through socialization so that learners are coached while exploring a suitable identity. 

Calibration. This structural attribute was distilled from nearly one-third of the articles. Its role and impact can be best explained from a social cognitive perspective; for instance, the Constructive-Developmental Theory of Self from Kegan [150]. This theory describes identity development following five successive stages. At each stage, individuals progress in their integration of cognitive, social and emotional capacities [150,151]. Kegan’s theory has been adopted by Bebeau and Monson [152] to fit an approach applicable for professional identity formation. They stress three stages that mirror the behavior of aspiring professionals and one additional stage that mirrors our view on IPI. Throughout these four stages, professionals gradually develop from adopting a narrow perspective on professional behavior to becoming ‘Moral Exemplars’. The latter implies becoming a high-functioning and strongly committed professional with leadership capabilities and the capacity to recognize interdependent systems and develop interdependent relations [86,104,152]. The resulting professional’s meaning-making of social reality has changed from a profession-centric to an interprofessional orientation through a calibration process [104,152]. This calibration refers to the role of one’s metacognition: a person’s professional awareness and accuracy of what they know and do not know and how well they are able to adapt to external demands [153]. The impact of calibration is manifested when knowledge and expertise improve by challenging existing beliefs and deconstructing previous ways of knowing to gain better fitting information from educational and life-experiences [151,154]. Hence, a calibration process depicts the learning trajectory when individuals construct step-by-step coherent values and beliefs that fit the required awareness, openness, self-efficacy and commitment that have been defined as the content-related attributes of IPI. Accordingly, this attribute introduces a focus on antecedents to consider when guiding learners to construct a belief system that aligns with their IPI. 

The transition into a ‘Moral Exemplar’, including the metacognitive calibration and dynamic cycles of exploration-commitment, is considered a lifelong process that extends far beyond higher education [152]. Interestingly, the emphasis on the former two structural attributes is mainly found in education-oriented articles, often articles which emphasize that professional identities are more difficult to change after graduation [102,155]. Nevertheless, even without an explicit educational intervention, professional identities continue to change through workplace socialization. This makes the focus on the dynamic development of IPI of high importance [139,156]. Similarly, this focus could also be adopted when looking at prospective students, already adopting a ‘pre-professional identity’, often already before enrolling in an education program. This is partly due to anticipatory socialization through life experiences, social media and interactions with relatives and friends who may or may not work in health or social care [145]. Although a focus on anticipatory and workplace socialization is largely absent in the reviewed literature, gaining an understanding of the influence of personal and contextual factors helps in discovering critical learning points in IPI development. It also emphasizes the need to look at the professional continuum and how the learning trajectory unfolds over time [68,69]. This continuum can act as a framework for organizing education that could help in tackling the impact of the ‘silos’ when learners are enrolled in undergraduate, graduate and continuing education and—when combined with IPE—the silos between educational programs [157]. Correspondingly, the professional continuum has been adopted as a priority frame of reference in addition to the instructional design approach by Valcke [158] to structure and discuss the synthesized antecedents in terms of the learning goals, content and instructional strategies related to the organization of a suitable learning environment as well as in terms of the conditions and contextual characteristics that enable IPI development. 

### 3.3. Antecedents

In line with the attributes described above, the main goals for developing an IPI are to adopt the necessary values, gain a greater social and self-awareness, cultivate an openness towards others, strengthen one’s self-efficacy, and to develop a commitment to collaborative relationships. Correspondingly, the antecedents shown in Table 1 are expected to be aligned with these goals. However, we opted for an alternative discussion of the antecedents as the individual research articles reviewed in our review study often centered on isolated antecedents, whereas our critical synthesis emphasizes how they are interrelated as they all describe how the personal experiences of learners and professionals are invoked, influenced and evaluated. This synthesis has led to two higher order themes which we will explore in the next paragraphs: constructivist learning environments (CLE) and intergroup leadership. The former integrates the antecedents related to curricula, educational strategies and role learning, while the latter is a synthesis of the following antecedents: breaking down barriers, intergroup leadership and community of practice. 

#### 3.3.1. Constructivist Learning Environments

In a CLE, learners are provided with the optimal conditions to improve their metacognitive competences and (re)structure their mental models so that a broader interprofessional orientation can develop and transformative learning may manifest [159]. To achieve this, a holistic view on learning has to be adopted by focusing on collaborative, constructive and contextual learning principles and through an emphasis on the cognitive, affective as well as social dimensions. In this way, a learner-centered environment can be created where learners actively and collaboratively construct their knowledge through the transformation of personal experiences under the guidance and scaffolding of teachers, faculty, tutors and coaches [160]. Accordingly, the following paragraphs have been structured in line with the three aforementioned learning principles. 

Collaborative learning. Collaborative learning is intrinsically linked to IPI-fitting CLE where learners from two or more professions construct meaning together by learning with, about and from each other in an interdependent way [110,158]. Through collaborative learning, existing beliefs are being evaluated and/or developed, leading to the construction or modification of knowledge structures associated with an IPI. The development of collaborative beliefs can therefore be stimulated by offering opportunities for formal and informal interactions. The majority of IPE initiatives found in the literature tend to be focused on these interactions and are often grounded in the Intergroup Contact Theory [82,93,105]. With this theory, Allport et al. [161] hypothesize that contact between groups reduces intergroup bias and prejudice as learners become acquainted with each other, thereby making it more likely that they develop relationships. Several educational strategies can be employed to foster such relationship building, for instance through cross-professional education, conferences, team-building, case studies and group projects [69,82,93,94]. These projects may be oriented towards tackling complex cases or solving problems, which is central within case-based learning (CBL) and problem-based learning (PBL) approaches. In CBL and PBL, learners share their prior knowledge in small interprofessional groups through collaborative inquiry so that the constructed knowledge can be applied to new and different contexts [157,162]. A similar yet more novel interdisciplinary learner-driven approach is challenge-based learning (ChBL). ChBL is oriented at addressing challenges of the 21st century and provides learners across health as well as non-health programs a framework with which they attempt to design and co-create a sustainable solution through the use of technology and collaboration with stakeholders from various settings [163]. 

Strategies such as CBL, PBL and ChBL prove to be especially effective in enhancing learner’s transversal competencies such as critical and creative thinking. However, they are also associated with an increasing complexity, which requires learners to become skilled in negotiation, shared decision-making and conflict management so that they are able to explore, articulate and share profession-specific knowledge and grow in the role of boundary spanners [83,94,96,158]. Associatively, as stated by Sargeant et al. [115] (p. 229), “contact is not enough to build respect and change stereotypes and long-held attitudes” as several other conditions that impact the quality of contact should be taken into account. For instance, bringing together learners with distinct professional identities, while assuming equal group status can, according to Nimmon et al. [164] (p. 247), be “ineffective because coercing individuals into intergroup interactions can reinforce stereotypes, especially when power relations that traverse health care’s professional hierarchy are obscured or ignored”. Hence, one of the main challenges in IPE is to emphasize the value of equality and foster the construction of beliefs that power is to be distributed flexibly, depending on the situation and expertise and not on the basis of traditions or the resulting inequal distribution of power [164,165]. According to Amerongen et al. [166], a promising approach to achieve relative equality between learners is by ensuring that the content of IPE is relevant for each participant, and that cases entail complementarity of professionals’ roles and perspectives and require equal task efforts and distribution to reach a shared goal. Little, however, is further known about educational strategies that enable these power dynamics. Nevertheless, this perspective is complementary to current social-psychological IPE underpinnings that ask for realistic learning situations in which a focus on knowledge, power and identity can be integrated [167,168]. The former is also related to the assumption that instructors should focus less on individual knowledge construction, but also on the active involvement of learners, the facilitation of critical reflection and the provision of opportunities for collaborative problem-solving [158]. 

Constructive learning. Constructive learning is essential in improving the quality of contact by engaging learners in conversation as well as facilitating articulation and reflection about their experiences so that they acquire a shared understanding of each other’s values [160]. This way, an awareness and openness develops with which learners are able to challenge and (re)construct their implicit beliefs and values related to collaborative practice [103]. For this reason, reflection should constitute a core aspect throughout the curriculum as challenging assumptions and refining beliefs and values should proceed in a sustained way by learners who act as reflective practitioners [169,170]. Reflective practice is essential for transformative learning, in which the organization of the self and the underlying knowledge structures are adjusted when being confronted with disorienting dilemmas or emerging crises [147]. In this case, the main role of instructors is to scaffold appropriately when dealing with such emerging crises by asking questions, stimulating reflection, giving authentic examples or explanations, and by letting learners paraphrase what they have learned [158,159]. Conversely, instructors may also choose to introduce cognitive conflicts such as common misconceptions and contradictory experiences in group discussions to activate prior knowledge and promote the critical evaluation of information from different perspectives so that existing views can be challenged and a co-construction of meaning can manifest [141,159]. 

However, a sufficient knowledge base and baseline in profession-related skills should first be developed before being expected to reflect on other professional roles. Learners might initially be unfamiliar with their own professional roles at the early development stages [171]. The risk of insufficient role knowledge is that confrontation with stereotypes may induce social anxiety, in which the contact is perceived as a threat to the identity that may exacerbate prejudice [166,172]. Addressing this knowledge insufficiency and social anxiety early enough is essential by gradually improving the knowledge and insights about roles, responsibilities, disciplinary boundaries, treatment models and communication practices with an emphasis on the similarities, differences, strengths and limitations [102,142]. Especially valuable is the learning about what differentiates and unites professionals as this may foster a greater awareness of existing assumptions and misconceptions, and the effects they might have [102,105]. This way, social anxiety may reduce while the readiness for collaborative learning and working increases [166]. Hence, constructive learning requires that a safe, confidential and trusting environment with enough space and time for reflection is established for learners to become familiar with the perspectives of others and to articulate ambiguous and conflicting experiences and ideas on equal footing [115]. Furthermore, a long-term focus across the professional continuum should be employed to prevent learners from bringing their stereotypes with them into practice or sustain them as practitioners [105,115]. Correspondingly, regular formal and informal cross-professional interactions with opportunities for critical reflection should be provided, for instance by organizing interprofessional speed-dating and various interprofessional modules [77,81,171]. These strategies can be supplemented with the use of media that support the scaffolding of reflection. One approach is through reflective writing, in which learners gain insight into who they are and who they aspire to become as professionals, for instance by conducting a thorough analysis of a significant event [169]. 

Contextualized learning. Contextualized learning plays an essential part in establishing a CLE as it allows learners to obtain experiential knowledge by actively constructing meaning through reflective interactions with realistic and authentic learning situations. In these situations, the learning context resembles or occurs in real life, thereby including contextual factors such as social, cultural and hierarchical influences. This way, learners develop a higher self-efficacy with which they become more able to tackle intellectual, social and motivational challenges [160,173]. An effective strategy is simulation-based education where learners are given the opportunity to actively improve their understanding of professionals’ roles while attempting to demonstrate their collaborative, leadership and problem-solving capabilities in a safe and controlled environment [174,175]. Simulations can take place face-to-face or online, make use of authentic cases and can be recorded, which proves invaluable for supervised debriefing sessions with peers to stimulate critical reflection and enhance their confidence [175,176]. Ideally, learners are sufficiently prepared before engaging in simulations, for instance through observation of video cases depicting modelled interprofessional behavior or by participating in workshops and skills labs where specific competences can be mastered after obtaining the necessary knowledge. In the case of the latter, near-peer tutoring may prove to be a powerful strategy, where tutors, who are quite similar in profile to the tutees but have progressed further in their training, can explain and model certain skills and behaviors for tutees to learn [120,121]. Conversely, simulations may be an appropriate steppingstone for more complex learning strategies such as situated learning, where learning occurs in the workplace, for instance through the work shadowing of professionals outside one’s field or through cross-professional mentorship [76,81]. More so, learners from different programs may take on primary responsibility in providing integral care in student-run units for a specific population such as at the orthopedics or emergency department, while being supervised and coached by multiple professional mentors [177]. Similarly, student-led clinics can be organized through a partnership with community-based organizations where service is provided to underserved populations, which requires the integration of multiple perspectives that strengthens learner’s social responsibility and civic engagement as well their cultural competences and collaborative skills [178]. 

The main benefit of these workplace-based activities is that learners gain hands-on experience in an authentic social context while benefiting from an individualized trajectory, in which they are scaffolded formally and informally in learning complex skills and strategies through a mutual and prolonged engagement [81,120]. During this trajectory, learners gradually develop their identity from being a newcomer to becoming a full participant of their professional CoP as well as of an interprofessional CoP [5,142]. In this interprofessional CoP, members progressively learn to navigate the landscape of CoPs, starting with observational learning towards gaining a greater knowledgeability and responsibility by learning with, from and about each other’s practices and expertise [158,179]. This learning by observation and doing is essential within contextualized learning for increasing learners’ understanding of each other’s roles and capabilities as well as ameliorating their self-efficacy through calibration [120,179]. Calibration can also be facilitated by means of feedback to reinforce positive behavior, stimulate reflection and offer the possibility to bond, discuss problems and share experiences [120,152]. As role knowledge insufficiencies and power dynamics may complicate effective feedback, developing literacy in interprofessional feedback is important so that the interprofessional team may become empowered to engage in a reciprocal process of feedback [179,180]. This way, a shared awareness, openness and commitment may develop that prompts a reappraisal of personal cognitions, resulting in a mutual understanding and acceptance through the co-construction of a TMS and a shared IPI [99,144]. 

#### 3.3.2. Intergroup Leadership

Leadership and institutional support at macro- and meso-levels is essential as the construction of sustainable CLE is dependent on several conditions and contextual characteristics that should be addressed [172]. Accordingly, leadership at the level of institutions and departments as well as a commitment from governance, management, faculty, champions and other stakeholders is paramount in dismantling professional silos and redesigning education as well as in constructing an interprofessional CoP that enables the development of IPIs [69,102]. 

Educational redesign. Essential in educational redesign is the futureproofing of curricula by adopting competency frameworks, such as that of the Interprofessional Education Collaborative (IPEC), so that learners are socialized to become T-shaped professionals capable and confident in their capabilities of collaborating with others [114,133]. In addition, we reiterate that developing an IPI requires the consolidation of experiences and the construction of belief systems based on evidence and reason [181]. Hence, a redesign also requires identifying and addressing the hidden curriculum, which refers to the informal learning activities from which learners develop implicit beliefs that do not necessarily align with the formal curriculum by adopting certain behaviors from role models and through day-to-day interactions. This curriculum allows for misconceptions and archaic beliefs, such as those related to the inequal distribution of power, to persist and to be passed on from generation to generation [182]. Therefore, the results of the present study push the idea to consider more strongly the importance of defining learning outcomes in terms of professionals beliefs that requires an appropriate approach in curriculum design, choice of didactics and use of assessment methods, for which we have provided suitable recommendations [183,184]. Although the precise timing of implementing these recommendations requires further research, we believe that establishing a culture of interprofessionalism that extends beyond higher education should occur as early as possible to promote the development of IPIs among learners as well as establish an interprofessional community where professional and faculty development is strengthened and intergroup leadership is emphasized [105,185]. 

Interprofessional CoP. Intergroup leadership is considered indispensable in facilitating a cultural change towards interprofessionalism and constructing an interprofessional CoP. Correspondingly, leaders who themselves have developed a strong IPI are in the ideal position to function as role models considering their experience and broad orientation [132,170]. Through intergroup leadership, followers from different backgrounds are influenced and motivated to contribute towards a shared organizational vision and common goals as well as supported in assuming informal collaborative leadership within their teams [127,136]. In other words, leaders impact the identity of professionals by representing the interests of the interprofessional CoP and by promoting a mutual commitment as a way of uniting professionals from distinct groups [9,127]. Nonetheless, functioning as a role model for members of every associated group may prove to be difficult, which requires forming a boundary-spanning coalition where leaders attempt to facilitate interaction between two or more professions by transferring the self-interest of their professional group to an interest in collaboration [7,11]. To achieve this, leaders need to connect and convince professionals by promoting an understanding of the shared goals and the important part they play in realizing them [186,187]. Connecting professionals is possible by breaking down barriers and structurally bringing people together in the same location, for instance through co-location and by organizing team meetings and teambuilding activities [82,92,93]. Creating a supportive, trusting and caring environment that corresponds with a CLE is warranted where colleagues learn to communicate and understand each other’s perspective so that their new identity, in which they view each other as ‘we’ rather than as ‘us versus them’, becomes normalized [186,188]. To sustain this identity, leaders have an important role in determining the perceptions, need for knowledge and other resources, and potential issues to proactively clear up misunderstandings and resolve conflicts through active observation and coaching of professionals [187,189]. Additionally, team-based rewards as well as developing an agreement containing mutually accepted norms and criteria of success could prevent conflicts and enhance team effectiveness [82,92,93]. Although the theory of intergroup leadership is relatively new in the domain of social psychology, it appears to be indispensable in explaining the intergroup dynamics evident in health and social care, for which further research is advised to empirically validate these insights in this context [11,128]. Additionally, adopting this theory together with the conceptual framework presented in this article may prove to be invaluable for future studies aimed at developing and evaluating complex educational interventions and its consequences. 

### 3.4. Consequences

The consequences related to the development of IPI are discernable at the levels of professional, team and health system. The most notable consequence at the individual level was professional wellbeing, in which professionals share a feeling of belonging and attachment, experience professional solidarity and fulfillment, are confident and motivated, and take pleasure in collaborating [76,83,87,103]. These outcomes fit well with those reported in the meta-analysis by Kaiser et al. [190], who conclude that IPC is crucial in achieving a collective wellbeing across professions as it facilitates an efficient and meaningful distribution of work demands, helps to achieve organizational goals, and stimulates personal growth as well as professional development [190,191]. In this case, the enactment of collaborative behavior may be attributable to the high level of social capital associated with IPI owing to the shared values, mutual trust as well as the collaborative approach in leadership. In turn, this behavior is supportive for IPI development, which may foster or further sustain an intrinsic motivation to collaborate [192]. At the team level, IPI has the potential to enhance team effectiveness, for instance due to effective communication, timely referrals and a high quality of decision-making that may be associated with the development of a TMM [82,92,93,94]. These outcomes are consistent with the meta-analysis of Zhou and Pazos [193], who posit that effective teams experience mutual trust and share a collective efficacy in the ability to attain organizational goals through unified efforts [142,193]. On this basis, we could argue that IPI is a condition that ensures that professional diversity within teams is inducive for team effectiveness as it supports complex problem-solving and enhances innovation by sharing knowledge and skills in an open-minded way and without feelings of hostility [143,186]. This way, congruent teams may ultimately lead to an improved health system performance that is more efficient, cost-effective, and patient-centered, and that leads to better health outcomes, increased patient satisfaction and a higher quality and safety of care [69,77,93,94]. Correspondingly, IPI can be considered a driver for interprofessional practice as well as a strategy to help achieve the quintuple aim of health care transformation through the improvement of patient experiences, population health, professional wellbeing, cost-effectiveness and health equity [191,194]. 

In spite of the promising outcomes linked with IPI, we re-emphasize the interpretative and hypothesis-generating nature of the analysis as it was beyond the scope of this study to consider the empirical evidence. However, we wish to stress the significant role IPI may play in the prediction of enacting collaborative behavior, which makes it indispensable to include in future studies that attempt to assess the outcomes of IPE and IPC, especially considering the scarce and mixed evidence on their effectiveness in terms of organizational change or patient outcomes [195,196]. This notwithstanding, recent reviews do seem to suggest growing and convincing evidence of IPE leading to coordinated patient-centered care and having a positive impact on patient and process outcomes [190,197,198,199]. If we wish to extend on this evidence base and become able to draw generalizable conclusions, then more rigorous research is required to determine which components work, when, why, and for whom in what circumstances. This may be attainable by adopting a suitable theoretical framework, clearly describing the format in terms of content and duration, focusing on relevant outcomes, and using validated measures that takes into consideration both individual- and team-level factors [196,199]. Importantly, future research should also take into account the potential moderating and mediating variables influencing the relation between IPI, the intention to collaborate as well as the resulting behavior [124,125]. Defining the empirical referents in terms of how to measure change in IPI may thus be useful in informing research and assessment practices. 

### 3.5. Empirical Referents

Assessment is essential in the creation of CLE to warrant a learner-centered curriculum across the professional continuum. Most fitting with a constructive pedagogy is programmatic assessment (PA). In this case, the assessment is a program in itself that aims to acquire rich and meaningful information of learners’ progression and their individual strengths and weaknesses in certain domains by means of an optimal mix of methods to use for formative as well as multi-source assessments [200]. This assessment for learning goes further than the more traditional assessment on learning that usually focusses on summative assessment, which tends to measure individual aspects of a construct and make use of behavioristic assessments of learners’ performance and output [200,201]. Considering that observed behavior does not necessarily provide information on the underlying processes, PA is deemed a more suitable approach if we wish to gain a compressive understanding of a learner’s IPI as this mostly develops in the mind, with some aspects only surfacing through verbal or non-verbal interactions [139,202]. Moreover, this social constructive pedagogy views assessment as inextricably embedded within learning activities, often termed assessment as learning. Here, the role of assessment is to promote intrinsic motivation as well as provide opportunities for multisource feedback and periodic critical reflections to encourage self-regulated learning along the professional continuum [201,203,204,205]. However, in view of the nascent research on IPI, little is further known about its assessment and the field of IPE has to advance to go beyond the surveying of learners’ attitudes to the development of a system of assessment that integrates different measures at multiple points in time and across different authentic contexts [205,206]. The choice in assessment approaches should also be oriented at the apex of Miller’s [183] pyramid, amended by Cruess et al. [13]. This means that assessment should go further than focusing on that what a learner ‘Does’ as a competent professional by assessing the acquisition of the learning outcomes at the highest ‘Is’ level, in which one develops an identity with which they come to think, act and feel like a professional. Al-Eraky and Marei [14] even propose adding an extra level where professionals are expected to ‘Do Together’, thereby inferring that assessment should cover both the individual and team level. 

Accordingly, strategies should encompass the assessment of values and beliefs indicative of an IPI through self-report questionnaires or team self-assessments, by analyzing reflective writing of essays, reports or any other (group) projects, or by conducting face-to-face open-ended interviews or focus groups [98,104,205]. The reviews by Peltonen et al. [207] and Jacob et al. [208] supply a useful overview of quantitative measures of IPC, although Peltonen et al. [207] shed light on the considerable overlap in content, lack of theoretical foundation, predominant focus on physicians and nurses, and variability in psychometric properties. For this reason, they emphasize the need for developing theoretically supported and generic instruments that are able to detect a responsiveness to change [207]. Qualitative analysis, on the other hand, of reflective writing may supply rich information of learner’s knowledge and beliefs about professional roles that may give an indication of their stage of identity development. Adopting interpretative phenomenology analysis and narrative interviews may be especially useful as they focus on the meaning of identity and provide information on how one makes sense of their identity [75,96,152]. The insights obtained from these quantitative and qualitative methods can also be triangulated with data collected through observation of collaborative behavior by means of standardized forms, group debriefings, placement evaluations, and so on [205]. Ideally, these assessments proceed in authentic learning environments and are performed by multiple evaluators to ensure a mix of perspectives that allow for formative multisource feedback and engage learners in self-and peer assessment [179,203,204,205]. The use of an e-portfolio may further facilitate and contribute to the assessment of IPI, in which learners digitally document a repertoire of evidence such as self-assessments, videos, projects, observations, evaluations and essays to demonstrate their learning progress and professional development [203,205,206]. E-portfolios are especially powerful considering the ownership that learners experience while constructing their personalized portfolio and the opportunities they receive to reflect on their learning trajectory under the scaffolding of a mentor or a coach [203,206,209]. Basu [203] also emphasizes the importance of including collaborative activities and peer feedback, for instance by sharing the portfolio with other students or staff, as this supports learners in identifying their strengths and weaknesses and integrating new information into their pre-existing knowledge [209]. As e-portfolios themselves require appropriate evaluation, rubrics can be developed that may be actively used by learners and assessors for self-and peer-assessment purposes [203,210]. A rubric is a scoring tool that can be used to assess a wide variety of assignments including external observations on the basis of prespecified criteria and performance indicators, which represent different levels of mastery [210]. Rubrics are valuable tools within CLE as they allow a reliable assessment of authentic learning while also acting as a guideline that articulates the expectations for learners’ work and behavior. This way, learners’ preparation, self-assessment and self-reflection is supported and possibilities for peer and multisource feedback can be offered [210,211]. Accordingly, a next step to advance the field of IPI could be the construction of rubrics through the co-involvement of learners and other stakeholders, which can be used to assess IPI development amongst learners with different professional backgrounds, in various settings, across the professional continuum, and by means of multisource feedback [211]. 

### 3.6. Strengths and Limitations

Several limitations to this study are to be noted. First of all, the CIS is a flexible and creative methodology in theory building, which inherently may increase the risk for researcher bias [16,19]. Appropriately, we supplemented our systematic search with additional purposeful searches and triangulated our perspectives in a reflexive way through regular discussions within our interdisciplinary team to guarantee confirmability and credibility of the results [212,213]. Furthermore, we attempted to maximize the agreement and consistency in our interpretations by means of a co-constructed coding book. An audit trail was developed to enhance dependability and trustworthiness, comprising the documentation of, for instance, memos, earlier versions, rationale for choice of methods, and meeting reports [212,214]. The combination of methodologies and adherence to the PRISMA and ENTREQ reporting guidelines further ensures transparency and reproducibility of study results [26,27,214]. However, in spite of our rigorous approach, the literature was scant, diverse and largely undertheorized, which inadvertently could have led to misinterpretations of the original researchers’ views on IPI, whom we did not directly contact for elaboration or verification [213]. This notwithstanding, we believe that theoretical saturation was reached considering our meticulous approach in selecting, analyzing and critically synthesizing the literature from different fields of research, and this broad inquiry led to the synthesis of both the content-related features and structural properties of IPI [139]. Associatively, the main strength of this study is that multiple components have been integrated into one broad perspective on identity that can be translated into structured educational programs, although one could argue whether some of these components represent distinct, complementary or associated aspects of IPI [215]. According to Berger and Lê Van [215], including different types of aspects in a broader perspective of identity is not uncommon and even preferred over treating components independently, which often is the case in the literature [125,145]. Nonetheless, we advise to regularly review the conceptualizations and uses of IPI in the literature as the meaning of concepts may evolve due to cultural, contextual and societal changes, prompting us to revisit or expand our interpretation of IPI [215,216]. Future studies may also validate the general fit of the model as well as determine the generalizability of our findings to contexts outside of the health and social care context, as our understanding of IPI may be equally relevant in other complex contexts considering the unequivocal need for a future workforce to collaborate interprofessionally as a way of tackling emerging societal challenges.

## 4. Conclusions

The purpose of this study was to identify, analyze and critically synthesize the assumptions and conceptions of IPI in the literature to construct a synthesizing argument and conceptual framework. This way, we were able to clarify what constitutes IPI, how and why it develops at the level of the individual and the team, as well as elucidate the conditions and contextual characteristics that enable IPI development and determine the potential outcomes. We posit that IPI infers that a professional’s meaning-making of collaborative practice develops from a profession-centric to a broader interprofessional orientation, in which one gains an awareness of their values and beliefs, acquires a greater openness towards collaboration, and feels increasingly confident in acting accordingly as a committed member of an interprofessional community. IPI is believed to potentially improve professional wellbeing, team effectiveness and ultimately contribute to reaching the quintuple aim through improved collaboration. Therefore, it is vital to develop constructivist learning environments that define learning outcomes in terms of professional beliefs and that make use of programmatic assessment to facilitate transformative learning across the professional continuum. Concurrently, a culture of interprofessionalism should be established, which extends beyond the boundaries of higher education and fosters the building of an interprofessional community of practice through intergroup leadership. To conclude, our conceptual framework may prove valuable to adopt in the (re)design, monitoring and quality control of educational programs and the evaluation of training effectiveness. More research is required to empirically validate our findings in different settings, to explore the underlying processes of IPI development, and to determine the interrelatedness between the attributes, the influence of group dynamics and other moderating or mediating factors. Future studies should also aim to develop effective assessment tools, help identify which components of instruction work, and implement our findings by means of instructional design principles.

## Figures and Tables

**Table 1:** Synthesis of attributes, antecedents and consequences of IPI (*n* = number of grouped articles from which results was extracted).

Antecedents	Attributes	Consequences
Interprofessional curricula, in which interprofessional competencies, longitudinal interventions and a focus on continuing education are integrated in addition to institutional support as well as a commitment across stakeholders with designated formal leaders (*n* = 8). Educational strategies that respect learners’ previous socialization processes and focus on identity development and reflection, in which students learn together in an interprofessional community of practice through workplace learning with cross-professional mentorship and collective learning activities with interactive teaching and feedback (*n*= 21). Interprofessional role learning by teaching students how to identify with both their own profession and the interprofessional community as well as support the development of a self as ‘boundary spanners’ in which students possess knowledge and understanding of other disciplines’ and perceive a sense of shared responsibilities, mutual aims and common goals (*n* = 15). Breaking down barriers with critical reflection upon assumptions towards others and challenging of misperceptions, stereotypes and prejudice by promoting an open attitude and understanding through exposure to interprofessional interactions in which self-awareness about the personal values and affinities for working and learning is fostered (*n* = 17). Intergroup leadership in which team members identify with leaders who employ a proactive and transformational leadership style, enable participative decision-making, and involve knowledge brokers as well as facilitate interprofessional openness and commitment while also managing conflicts and nurturing consensus (*n* = 7). An interprofessional community of practice that facilitates knowledge transfer and is characterized with regular formal and informal interprofessional interactions in addition to the presence of team-based rewards, mutually accepted norms, and team-based criteria and procedures for communication and treatments (*n* = 9)	Interprofessional values for collaboration which may relate to having a moral responsibility of collaboration, acknowledging the interdependency between professionals, desiring an equal distribution of power based on relevant expertise, feeling a sense of togetherness through mutual involvement, respecting other professionals’ expertise and trusting each other to share expertise, opinions and feelings (*n* = 20). Interprofessional awareness of any preconceived assumptions about other professionals in terms of stereotypes, similarities and differences between professionals as well as being convinced about the value of collaboration (*n* = 8) Interprofessional openness by valuing and being willing to actively share their own and involve a diversity of other professionals’ perspectives (*n* = 10). Interprofessional self-efficacy by being confident and feeling comfortable in the own capabilities for interprofessional learning and working such as those related to professionals’ roles, leadership, teamwork and communication (*n* = 28). Interprofessional commitment characterized by a sense of belonging to an overarching interprofessional community and a self-view of being an accepted member that takes on the role of interprofessional (*n* = 24). Context-dependent as IPI becomes salient when there is a fit with a situation that requires an interprofessional approach (*n* = 11) Team mental model in which one feels part of an interprofessional team where everyone works side-by-side and seem to have a joint commitment and a shared view on how to collaborate in an adaptable, responsive and consistent way (*n* = 8) A fluid and dynamic developmental course through negotiated experiences and consideration of possible alternative identities (*n* = 15) Calibration in which a more advanced way of meaning-making pertaining collaborative practice has developed through re-interpretation of experiences (*n* = 9)	Professional wellbeing in which one feels motivated, confident, satisfied and with a good mood in addition to feeling valued, empowered, responsible and integrated in the team as well as perceiving a sense of fulfillment, freedom and team cohesiveness (*n* = 12) Team effectiveness, characterized with team communication, effectiveness, and solidarity in which there is clarity about own and other professionals’ roles as well as experiencing a positive team culture with understanding and trust, in which collaborative behavior is demonstrated that leads to better knowledge transfer and decision-making (*n* = 15) Health system performance as interprofessional collaboration boosts cost-effectiveness and innovation as well as improves population health and patients’ experiences through patient-centered, safe and high quality care, in addition to improved recruitment and retention due to high professional wellbeing (*n* = 7)

## Data Availability

The data presented in this study are available in the Supplementary Material.

## Supplementary Materials

The following supporting information can be downloaded at: https://www.mdpi.com/article/10.3390/ijerph192214799/s1, all data generated or analyzed during this study are included in this published article and following supplementary information files; Table S1: Vignettes; Table S2: Reporting Checklist; Table S3: Search strings; Table S4: List of excluded studies; Table S5: Data-extraction tables; Table S6: Code book. Refs. [31,32,33,34,35,36,37,38,39,40,41,42,43,44,45,46,47,48,49,50,51,52,53,54,55,56,57,58,59,60,61,62,63,64,65,66,67,68,69,70,71,72,73,74,75,76,77,78,79,80,81,82,83,84,85,86,87,88,89,90,91,92,93,94,95,96,97,98,99,100,101,102,103] are cited in the supplementary files.
