# Supplementary material for: Interprofessional Identity in Health and Social Care: Analysis and Synthesis of the Assumptions and Conceptions in the Literature
Source: Int J Environ Res Public Health. 2022 Nov 10;19(22):14799. doi: 10.3390/ijerph192214799 (PMC9690615; doi:10.3390/ijerph192214799)
Supplement: Supplementary file 1 [file ijerph-19-14799-s001.zip › Table S1 - Vignettes.pdf]

Table S1

Educational vignettes describing short stories of a student or professional with a clearly, somewhat or not (clearly) developing interprofessional identity (IPI)

**Presence of a developing IPI**	**Student or professional**	**Vignette**
Clearly present developing IPI	Student	Simon is currently in his fifth week clinical placement at an Oncology department, which he finds both challenging and enjoying. The complex clinical presentations and apparent psychosocial needs has lead Simon to feel he has a moral responsibility of involving other disciplines to deliver good quality care[1]. He admits that this was easier said then done as a junior physician as he didn't feel prepared and secure at the beginning, although he lately perceives himself to have grown quite confident in his communicative and shared-decision making skills during the interprofessional rounds[2]. During these rounds, Simon has grown a habit to ask for feedback on his treatment recommendations and to actively ask others for their professional opinion[3]. He mostly values the positive experiences that he had with the psychologists and the social workers, which significantly changed his perceptions about the value of collaboration as he did not really understand the benefits they could bring to the table when he started of his education[4]. Now, he can proudly say he grown to be quite adept in the role of collaborator[5]. Simon has one week left for his placement to end and has gained quite a good notion of what his strengths and areas for growth are in relation to collaboration[6]. This placement, where he truly felt part of a cohesive team, has had such an impact on him that he decided to apply for an Internal Medicine residency where he hopes to encounter the same interprofessional challenges with his colleagues side-by-side[7].<br><br>[1]Values, [2]Self-efficacy, [3]Openness, [4]Awareness, [5]Commitment, [6]Calibration, [7]Team Mental Model
	Professional	As an Advanced Practice Nurse in wound care with 12 years of experience, Mark has had countless of experiences with interprofessional working that made him aware how important other disciplines' contributions are in his practice[1]. Throughout these years he has managed to become recognized in his expertise and to be well known throughout the hospital and the larger interprofessional community as a

		devoted practitioner[2]. He radiates a confident stance and acts as a leader who is regarded a role model that inspires team members to contribute to a shared purpose[3]. Mark is keen to actively share his knowledge and expertise with colleagues[4] whom he perceives to be on the same page as him[5]. More so, he is a pioneer who has managed to transform his wound clinic into an interdisciplinary center of excellence, in which he emphasizes the interdependent nature of treating wounds[6]. In line with the vision of his center, he has developed a broad focus[7] by involving a diversity of relevant perspectives to facilitate optimal wound healing, organize practical and financial support, improve dietary intake and ensure adequate pain management[4].<br><br>[1]Awareness, [2]Commitment, [3]Self-efficacy, [4]Openness, [5]Team Mental Model, [6]Values, [7]Calibration,
Somewhat present developing IPI	Student	Indra is almost halfway her last semester of placements leading to her graduation as a social worker. Her best experiences so far have been in the hospital where she guided adult patients who received cardio surgery to resume occupational activities after rehabilitation as a transition coach. During this placement she came into contact with different professionals during the weekly interdisciplinary meeting, whose contributions she valued and from which she gained a better understanding of what everyone's expertise is[1]. Although Indra is certain that every professional has an important contribution in the process, she thinks its more than enough to just keep informing everyone what they are doing[2]. After all, she feels its logical that social workers are trained well enough to independently organize their work and that everyone should trust and respect that[3]. She shares this mindset with her mentors and the other social workers with whom she feels a strong connection to the social work profession[4]. Even though everything appears to run smoothly for the social workers, Indra found out through discussion with a nurse that the other disciplines don't seem to fully agree. They believe the social workers' communication to be largely one-sided and would like to be involved more in a proactive way[5]. This news has been bothering Indra and she does not really know how to proceed with it and whether she should take it into consideration[6]. It also left her rather conflicted at moments and made her feel somewhat less confident in her communication with other professionals[7].<br><br>[1]Awareness, [2]Openness, [3]Values, [4]Commitment, [5]Team Mental Model, [6]Calibration, [7]Self-efficacy
	Professional	Angela has been working as an independent physiotherapist in a small town for the past 10 years. She loved her job with passion but decided she wanted some change in her career and applied for a job in a interdisciplinary sports center in the city nearby where she wants broaden her horizon and explore new opportunities[1]. Recent life-experiences also led her to look for more connections and to value trusting relationships more[2]. For this reason, she wants to feel a part of a bigger community of practitioners,

		although she believes she needs to learn a lot to become a full member considering her limited experience with assessing and treating complex sport injuries typical in this center[3]. Angela is quite eager to learn and is especially interested in getting to know everyone's' role and share experiences[4]. She admits that she is largely unaware and a bit skeptical of how the day-to-dare interactions between professionals may go as she remembers some of the communication issues she had with physicians over the phone in her own practice[5]. This makes Angela aware that she should work some more on her self-confidence the coming months[6]. Nonetheless, she is quite positive minded and already feels part of the team now that she has been selected, although she sometimes wonders how well they will all get along[7].<br><br>[1]Calibration, [2]Values, [3]Commitment, [4]Openness, [5]Awareness, [6]Self-efficacy, [7]Team Mental Model
Not (clearly) present developing IPI	Student	Richard has started his pharmaceutical education two years ago with the support of his parents, of which both work as drug developers in a big pharmaceutical company. Both wish their son grows up to be an excellent pharmacist, and preferably in a similar working setting as them. Richard was raised with the traditional values of diligence and perseverance which he constantly keeps in mind[1]. As a freshly enrolled student in his third year, he becomes more and more familiar with the roles of other disciplines in healthcare through his courses, although he doesn't really understand why this is part of the curriculum[2]. He believes that it is more important to learn about becoming a pharmacist as he wants to please his parents and be part of that same community[3]. Richard recently participated in an interprofessional course with medical and physiotherapy students, but did not really enjoy the group project. He decided for the group that it was best for each one to prepare and present their part and that it was not really efficient to share and discuss matters beforehand[4]. For him it was quite a surprise that the presentation ended up being mediocre at best, while it was clear for the assessors that there was a lack of team engagement[5]. Richard was offered the opportunity to improve his grade by the end of the semester, but he did not feel confident in re-establishing relations with his group members[6]. He rather wants to focus on improving his clinical skills to prepare him for his placements instead of considering the feedback he received[7].<br><br>[1]Values, [2]Awareness, [3]Commitment, [4]Openness, [5]Team Mental Model, [6]Self-efficacy, [7]Calibration,
	Professional	Sarah has been a resident at a rural hospital's radiotherapy department for around three years now. She has always been fascinated by the technological side of medicine and is passionate to apply innovative methods in the care for oncology patients. The sky is the limit for her and she works hard everyday to follow her dreams in becoming world renowned within the community of radiotherapists[1]. She has a strong desire to be one of the best in her fields and feel she should be treated accordingly by other

disciplines whom she feels matter less in the treatment of cancers[2]. This feeling is partly supported by her lack of knowing what the nurses, psychologists and other workers actually contribute in the complex care associated with these conditions[3]. Sarah also isn't exactly keen to discuss her treatment plans with her colleagues as she is convinced of her own expertise and doesn't want to invest in conversations with others[4]. She likes to view herself as an independent worker and doesn't like it when team members disturb her[5]. Sarah has received several comments from the head nurses and colleagues about her way of working and the suggestion to collaborate more often, but she doesn't really feel comfortable to do so[6]. Overall, Sarah tries to stay out of interactions with other disciplines and prefers to keeping focussing on her specific domain[7].

[1]Commitment, [2]Values, [3]Awareness, [4]Openness, [5]Team Mental Model, [6]Self-efficacy, [7]Calibration,
