# Supplementary material for: Interprofessional Identity in Health and Social Care: Analysis and Synthesis of the Assumptions and Conceptions in the Literature
Source: Int J Environ Res Public Health. 2022 Nov 10;19(22):14799. doi: 10.3390/ijerph192214799 (PMC9690615; doi:10.3390/ijerph192214799)
Supplement: Supplementary file 1 [file ijerph-19-14799-s001.zip › Table S2 - Reporting Checklist.pdf]

Table S2

PRISMA and ENTREQ Reporting Checklist

Section and Topic	Item	Checklist item (PRISMA)	Section and Topic	Guide and description (ENTREQ)	Location where items are reported
**TITLE**					
Title	1	Identify the report as a systematic review.			1
**ABSTRACT**					
Abstract	2	See the PRISMA 2020 for Abstracts checklist.			2
**INTRODUCTION**					
Rationale	3	Describe the rationale for the review in the context of existing knowledge.	Aim	State the research question the synthesis addresses	3-4
Objectives	4	Provide an explicit statement of the objective(s) or question(s) the review addresses.			
**METHODS**					
Eligibility criteria	5	Specify the inclusion and exclusion criteria for the review and how studies were grouped for the syntheses.	Inclusion criteria	Specify the inclusion/exclusion criteria (e.g. in terms of population, language, year limits, type of publication, study type).	7-8
Information sources	6	Specify all databases, registers, websites, organisations, reference lists and other sources searched or consulted to identify studies. Specify the date when each source was last searched or consulted.	Data sources	Describe the information sources used (e.g. electronic databases (MEDLINE, EMBASE, CINAHL, psychINFO, Econlit), grey literature databases (digital thesis, policy reports), relevant organisational websites, experts, information specialists, generic web searches (Google Scholar), hand searching, reference lists) and when the searches were conducted; provide the rationale for using the data sources.	7

			Approach to searching	Indicate whether the search was pre-planned (comprehensive search strategies to seek all available studies) or iterative (to seek all available concepts until theoretical saturation is achieved).	7-8
Search strategy	7	Present the full search strategies for all databases, registers and websites, including any filters and limits used.	Electronic Search strategy	Describe the literature search (e.g. provide electronic search strategies with population terms, clinical or health topic terms, experiential or social phenomena related terms, filters for qualitative research and search limits).	7 and Table S3
Selection process	8	Specify the methods used to decide whether a study met the inclusion criteria of the review, including how many reviewers screened each record and each report retrieved, whether they worked independently, and if applicable, details of automation tools used in the process.	Study screening methods	Describe the process of study screening and sifting (e.g. title, abstract and full text review, number of independent reviewers who screened studies)	8
Data collection process	9	Specify the methods used to collect data from reports, including how many reviewers collected data from each report, whether they worked independently, any processes for obtaining or confirming data from study investigators, and if applicable, details of automation tools used in the process.	Appraisal items	State the tools, frameworks and criteria used to appraise the studies or selected findings (e.g. Existing tools: CASP, QARI, COREQ, Mays and Pope [25]; reviewer developed tools; describe the domains assessed: research team, study design, data analysis and interpretations, reporting).	9
Data items	10a	List and define all outcomes for which data were sought. Specify whether all results that were compatible with each outcome domain in each study were sought (e.g. for all measures, time points, analyses), and if not, the methods used to decide which results to collect.	Study characteristics	Present the characteristics of the included studies (e.g. year of publication, country, population, number of participants, data collection, methodology, analysis, research questions).	9 and Table S5
	10b	List and define all other variables for which data were sought (e.g. participant and intervention characteristics, funding sources). Describe any assumptions made about any missing or unclear information.			
Study risk of bias assessment	11	Specify the methods used to assess risk of bias in the included studies, including details of the tool(s) used, how many reviewers assessed each study and whether they worked independently,	Rationale for appraisal	Describe the rationale and approach used to appraise the included studies or selected findings (e.g. assessment of conduct (validity and robustness), assessment of reporting (transparency), assessment of content and utility	10

		and if applicable, details of automation tools used in the process.	Appraisal process	Indicate whether the appraisal was conducted independently by more than one reviewer and if consensus was required.	10
Effect measures	12	Specify for each outcome the effect measure(s) (e.g. risk ratio, mean difference) used in the synthesis or presentation of results.			N/A
Synthesis methods	13a	Describe the processes used to decide which studies were eligible for each synthesis (e.g. tabulating the study intervention characteristics and comparing against the planned groups for each synthesis (item #5)).	Synthesis methodology	Identify the synthesis methodology or theoretical framework which underpins the synthesis, and describe the rationale for choice of methodology (e.g. meta-ethnography, thematic synthesis, critical interpretive synthesis, grounded theory synthesis, realist synthesis, meta-aggregation, meta-study,	5-6 and 9-10
	13b	Describe any methods required to prepare the data for presentation or synthesis, such as handling of missing summary statistics, or data conversions.			N/A
	13c	Describe any methods used to tabulate or visually display results of individual studies and syntheses.	Data extraction	Indicate which sections of the primary studies were analysed and how were the data extracted from the primary studies? (e.g. all text under the headings “results /conclusions” were extracted electronically and entered into a computer	9-10
	13d	Describe any methods used to synthesize results and provide a rationale for the choice(s). If meta-analysis was performed, describe the model(s), method(s) to identify the presence and extent of statistical heterogeneity, and software package(s) used.	Number of reviewers<br>Coding<br>Software<br>Derivation of themes<br>Study comparison	Identify who was involved in coding and analysis.<br>Describe the process for coding of data (e.g. line by line coding to search for concepts).<br>State the computer software used, if any.<br>Explain whether the process of deriving the themes or constructs was inductive or deductive.<br>Describe how were comparisons made within and across studies (e.g. subsequent studies were coded into pre-existing concepts, and new concepts were created when deemed necessary).	9-11
	13e	Describe any methods used to explore possible causes of heterogeneity among study results (e.g. subgroup analysis, meta-regression).			11

	13f	Describe any sensitivity analyses conducted to assess robustness of the synthesized results.			N/A
Reporting bias assessment	14	Describe any methods used to assess risk of bias due to missing results in a synthesis (arising from reporting biases).			11
Certainty assessment	15	Describe any methods used to assess certainty (or confidence) in the body of evidence for an outcome.			11
**RESULTS**					
Study selection	16 a	Describe the results of the search and selection process, from the number of records identified in the search to the number of studies included in the review, ideally using a flow diagram.	Study selection results	Identify the number of studies screened and provide reasons for study exclusion (e.g. for comprehensive searching, provide numbers of studies screened and reasons for exclusion indicated in a figure/flowchart; for iterative searching describe reasons for study exclusion and inclusion based on modifications t the research question and/or contribution to theory	7-8 and Figure 1
	16 b	Cite studies that might appear to meet the inclusion criteria, but which were excluded, and explain why they were excluded.	Appraisal results	Present results of the quality assessment and indicate which articles, if any, were weighted/excluded based on the assessment and	8 and Table S4
Study characteristics	17	Cite each included study and present its characteristics.			
Risk of bias in studies	18	Present assessments of risk of bias for each included study.	Quotations	Provide quotations from the primary studies to illustrate themes/constructs, and identify whether the quotations were participant quotations or the author’s interpretation	9-11 and Table S5
Results of individual studies	19	For all outcomes, present, for each study: (a) summary statistics for each group (where appropriate) and (b) an effect estimate and its precision (e.g. confidence/credible interval), ideally using structured tables or plots.			

Results of syntheses	20a	For each synthesis, briefly summarise the characteristics and risk of bias among contributing studies.			
	20b	Present results of all statistical syntheses conducted. If meta-analysis was done, present for each the summary estimate and its precision (e.g. confidence/credible interval) and measures of statistical heterogeneity. If comparing groups, describe the direction of the effect.	Synthesis output	Present rich, compelling and useful results that go beyond a summary of the primary studies (e.g. new interpretation, models of evidence, conceptual models, analytical framework, development of a new theory or construct).	12-37
	20c	Present results of all investigations of possible causes of heterogeneity among study results.			
	20d	Present results of all sensitivity analyses conducted to assess the robustness of the synthesized results.			
Reporting biases	21	Present assessments of risk of bias due to missing results (arising from reporting biases) for each synthesis assessed.			
Certainty of evidence	22	Present assessments of certainty (or confidence) in the body of evidence for each outcome assessed.			
**DISCUSSION**					
Discussion	23a	Provide a general interpretation of the results in the context of other evidence.			12-37
	23b	Discuss any limitations of the evidence included in the review.			40-41
	23c	Discuss any limitations of the review processes used.			40-41
	23d	Discuss implications of the results for practice, policy, and future research.			12-37
**OTHER INFORMATION**					
Registration and protocol	24a	Provide registration information for the review, including register name and registration number, or state that the review was not registered.			N/A
	24b	Indicate where the review protocol can be accessed, or state that a protocol was not prepared.			N/A

	24c	Describe and explain any amendments to information provided at registration or in the protocol.	N/A
Support	25	Describe sources of financial or non-financial support for the review, and the role of the funders or sponsors in the review.	Author’s note
Competing interests	26	Declare any competing interests of review authors.	Author’s note
Availability of data, code and other materials	27	Report which of the following are publicly available and where they can be found: template data collection forms; data extracted from included studies; data used for all analyses; analytic code; any other materials used in the review.	Author’s note
