# Supplementary material for: Interprofessional Identity in Health and Social Care: Analysis and Synthesis of the Assumptions and Conceptions in the Literature
Source: Int J Environ Res Public Health. 2022 Nov 10;19(22):14799. doi: 10.3390/ijerph192214799 (PMC9690615; doi:10.3390/ijerph192214799)
Supplement: Supplementary file 1 [file ijerph-19-14799-s001.zip › Table S3 - Search strings.pdf]

Table S3

Overview of used syntax for the second and third literature search (n=number of search results)

Database	Syntax January 2020	n	Syntax July 2020	n
PubMed	"multi-disciplinary identit*"[Title/Abstract] OR "multidisciplinary identit*"[Title/Abstract] OR "cross-disciplinary identit*"[Title/Abstract] OR "collaborative identit*"[Title/Abstract] OR "interdisciplinary identit*"[Title/Abstract] OR "interprofessional identit*"[Title/Abstract] OR "inter-professional identit*"[Title/Abstract] OR "interdisciplinary identit*"[Title/Abstract] OR "inter-disciplinary identit*"[Title/Abstract] OR "transdisciplinary identit*"[Title/Abstract] OR "trans-disciplinary identit*"[Title/Abstract] OR "intergroup relational identit*"[Title/Abstract] OR "team identit*"[Title/Abstract] OR "dual identit*"[Title/Abstract]	127	"reconfigured professional identit*"[Title/Abstract] OR "Care unit identit*"[Title/Abstract] OR "Superordinate identit*"[Title/Abstract] OR "Common identit*"[Title/Abstract] OR "Crossed identit*"[Title/Abstract] OR "interprofessional role identit*" [Title/Abstract] OR "extended professional identit*" [Title/Abstract] OR "group identit*"[Title/Abstract] OR "social identit*"[Title/Abstract] OR "collective identit*"[Title/Abstract] OR "multi-disciplinary identit*"[Title/Abstract] OR "multidisciplinary identit*"[Title/Abstract] OR "cross-disciplinary identit*"[Title/Abstract] OR "collaborative identit*"[Title/Abstract] OR "interdisciplinary identit*"[Title/Abstract] OR "interprofessional identit*"[Title/Abstract] OR "inter-professional identit*"[Title/Abstract] OR "interdisciplinary identit*"[Title/Abstract] OR "inter-disciplinary identit*"[Title/Abstract] OR "transdisciplinary identit*"[Title/Abstract] OR "trans-disciplinary identit*"[Title/Abstract] OR "intergroup relational identit*"[Title/Abstract] OR "team identit*"[Title/Abstract] OR "dual identit*"[Title/Abstract]	2.761
Web of Science	TI=("multi-disciplinary identit*" OR "multidisciplinary identit*" OR "cross-disciplinary identit*" OR "collaborative	147	TI=("reconfigured professional identit*" OR "Care unit identit*" OR "Superordinate identit*" OR "Common identit*" OR "Crossed identit*" OR "interprofessional	3,903

	identit*” OR “interdisciplinary identit*” OR “interprofessional identit*” OR “inter-professional identit*” OR “interdisciplinary identit*” OR “inter-disciplinary identit*” OR “transdisciplinary identit*” OR “trans-disciplinary identit*” OR “intergroup relational identit*” OR “team identit*” OR “dual identit*”)		role identit*” OR “extended professional identit*” OR “group identit*” OR “social identit*” OR “collective identit*” OR “multi-disciplinary identit*” OR “multidisciplinary identit*” OR “cross-disciplinary identit*” OR “collaborative identit*” OR “interdisciplinary identit*” OR “interprofessional identit*” OR “inter-professional identit*” OR “interdisciplinary identit*” OR “inter-disciplinary identit*” OR “transdisciplinary identit*” OR “trans-disciplinary identit*” OR “intergroup relational identit*” OR “team identit*” OR “dual identit*”)	
Scopus	TITLE-ABS-KEY("multi-disciplinary identit*" OR "multidisciplinary identit*" OR "cross-disciplinary identit*" OR "collaborative identit*" OR "interdisciplinary identit*" OR "interprofessional identit*" OR "inter-professional identit*" OR "interdisciplinary identit*" OR "inter-disciplinary identit*" OR "transdisciplinary identit*" OR "trans-disciplinary identit*" OR "intergroup relational identit*" OR "team identit*" OR "dual identit*")	731	TITLE-ABS-KEY(“reconfigured professional identit*” OR “Care unit identit*” OR “Superordinate identit*” OR “Common identit*” OR “Crossed identit*” OR “interprofessional role identit*” OR “extended professional identit*” OR “group identit*” OR “social identit*” OR “collective identit*” OR "multi-disciplinary identit*" OR "multidisciplinary identit*" OR "cross-disciplinary identit*" OR "collaborative identit*" OR "interdisciplinary identit*" OR "interprofessional identit*" OR "inter-professional identit*" OR "interdisciplinary identit*" OR "inter-disciplinary identit*" OR "transdisciplinary identit*" OR "trans-disciplinary identit*" OR "intergroup relational identit*" OR "team identit*" OR "dual identit*")	19,980
Embase	'multi-disciplinary identit*':ab,ti OR 'multidisciplinary identit*':ab,ti OR 'cross-disciplinary identit*':ab,ti OR 'collaborative identit*':ab,ti OR 'interprofessional identit*':ab,ti OR 'inter-professional identit*':ab,ti OR 'interdisciplinary identit*':ab,ti OR 'inter-disciplinary identit*':ab,ti OR	140	‘reconfigured professional identit*’:ab,ti OR ‘Care unit identit*’:ab,ti OR ‘Superordinate identit*’:ab,ti OR ‘Common identit*’:ab,ti OR ‘Crossed identit*’:ab,ti OR ‘interprofessional role identit*’:ab,ti OR ‘extended professional identit*’:ab,ti OR ‘group identit*’:ab,ti OR ‘social identit*’:ab,ti OR ‘collective identit*’:ab,ti OR 'multi-disciplinary identit*':ab,ti OR 'multidisciplinary identit*':ab,ti OR 'cross-disciplinary identit*':ab,ti OR	2.704

	'transdisciplinary identit*':ab,ti OR 'trans-disciplinary identit*':ab,ti OR 'intergroup relational identit*':ab,ti OR 'team identit*':ab,ti OR 'dual identit*':ab,ti		'collaborative identit*':ab,ti OR 'interprofessional identit*':ab,ti OR 'inter-professional identit*':ab,ti OR 'interdisciplinary identit*':ab,ti OR 'inter-disciplinary identit*':ab,ti OR 'transdisciplinary identit*':ab,ti OR 'trans-disciplinary identit*':ab,ti OR 'intergroup relational identit*':ab,ti OR 'team identit*':ab,ti OR 'dual identit*':ab,ti	
ERIC	ab("multi-disciplinary identit*" OR "multidisciplinary identit*" OR "cross-disciplinary identit*" OR "collaborative identit*" OR "interdisciplinary identit*" OR "interprofessional identit*" OR "inter-professional identit*" OR "interdisciplinary identit*" OR "inter-disciplinary identit*" OR "transdisciplinary identit*" OR "trans-disciplinary identit*" OR "intergroup relational identit*" OR "team identit*" OR "dual identit*") OR ti("multi-disciplinary identit*" OR "multidisciplinary identit*" OR "cross-disciplinary identit*" OR "collaborative identit*" OR "interdisciplinary identit*" OR "interprofessional identit*" OR "inter-professional identit*" OR "interdisciplinary identit*" OR "inter-disciplinary identit*" OR "transdisciplinary identit*" OR "trans-disciplinary identit*" OR "intergroup relational identit*" OR "team identit*" OR "dual identit*")	59	ab(“reconfigured professional identit*” OR “Care unit identit*” OR “Superordinate identit*” OR “Common identit*” OR “Crossed identit*” OR “interprofessional role identit*” OR “extended professional identit*” OR “group identit*” OR “social identit*” OR “collective identit*” OR "multi-disciplinary identit*" OR "multidisciplinary identit*" OR "cross-disciplinary identit*" OR "collaborative identit*" OR "interdisciplinary identit*" OR "interprofessional identit*" OR "inter-professional identit*" OR "interdisciplinary identit*" OR "inter-disciplinary identit*" OR "transdisciplinary identit*" OR "trans-disciplinary identit*" OR "intergroup relational identit*" OR "team identit*" OR "dual identit*") OR ti("multi-disciplinary identit*" OR "multidisciplinary identit*" OR "cross-disciplinary identit*" OR "collaborative identit*" OR "interdisciplinary identit*" OR "interprofessional identit*" OR "inter-professional identit*" OR "interdisciplinary identit*" OR "inter-disciplinary identit*" OR "transdisciplinary identit*" OR "trans-disciplinary identit*" OR "intergroup relational identit*" OR "team identit*" OR "dual identit*")	1.688

Cinahl	TI (“multi-disciplinary identit*” OR “multidisciplinary identit*” OR “cross-disciplinary identit*” OR “collaborative identit*” OR “interdisciplinary identit*” OR “interprofessional identit*” OR “inter-professional identit*” OR “interdisciplinary identit*” OR “inter-disciplinary identit*” OR “transdisciplinary identit*” OR “trans-disciplinary identit*” OR “intergroup relational identit*” OR “team identit*” OR “dual identit*”) OR AB (“multi-disciplinary identit*” OR “multidisciplinary identit*” OR “cross-disciplinary identit*” OR “collaborative identit*” OR “interdisciplinary identit*” OR “interprofessional identit*” OR “inter-professional identit*” OR “interdisciplinary identit*” OR “inter-disciplinary identit*” OR “transdisciplinary identit*” OR “trans-disciplinary identit*” OR “intergroup relational identit*” OR “team identit*” OR “dual identit*”)	93	TI (“reconfigured professional identit*” OR “Care unit identit*” OR “Superordinate identit*” OR “Common identit*” OR “Crossed identit*” OR “interprofessional role identit*” OR “extended professional identit*” OR “group identit*” OR “social identit*” OR “collective identit*” OR “multi-disciplinary identit*” OR “multidisciplinary identit*” OR “cross-disciplinary identit*” OR “collaborative identit*” OR “interdisciplinary identit*” OR “interprofessional identit*” OR “inter-professional identit*” OR “interdisciplinary identit*” OR “inter-disciplinary identit*” OR “transdisciplinary identit*” OR “trans-disciplinary identit*” OR “intergroup relational identit*” OR “team identit*” OR “dual identit*”) OR AB (“multi-disciplinary identit*” OR “multidisciplinary identit*” OR “cross-disciplinary identit*” OR “collaborative identit*” OR “interdisciplinary identit*” OR “interprofessional identit*” OR “inter-professional identit*” OR “interdisciplinary identit*” OR “inter-disciplinary identit*” OR “transdisciplinary identit*” OR “trans-disciplinary identit*” OR “intergroup relational identit*” OR “team identit*” OR “dual identit*”)	639
PsycArticles	ab("multi-disciplinary identit*" OR "multidisciplinary identit*" OR "cross-disciplinary identit*" OR "collaborative identit*" OR "interdisciplinary identit*" OR "interprofessional identit*" OR "inter-professional identit*" OR "interdisciplinary identit*" OR "inter-disciplinary identit*" OR "transdisciplinary identit*" OR "trans-	16	ab(“reconfigured professional identit*” OR “Care unit identit*” OR “Superordinate identit*” OR “Common identit*” OR “Crossed identit*” OR “interprofessional role identit*” OR “extended professional identit*” OR “group identit*” OR “social identit*” OR “collective identit*” OR "multi-disciplinary identit*" OR "multidisciplinary identit*" OR "cross-disciplinary identit*" OR "collaborative identit*" OR "interdisciplinary identit*" OR "interprofessional identit*"	497

	disciplinary identit*" OR "intergroup relational identit*" OR "team identit*" OR "dual identit*") OR ti("multi-disciplinary identit*" OR "multidisciplinary identit*" OR "cross-disciplinary identit*" OR "collaborative identit*" OR "interdisciplinary identit*" OR "interprofessional identit*" OR "inter-professional identit*" OR "interdisciplinary identit*" OR "inter-disciplinary identit*" OR "transdisciplinary identit*" OR "trans-disciplinary identit*" OR "intergroup relational identit*" OR "team identit*" OR "dual identit*")	OR "inter-professional identit*" OR "interdisciplinary identit*" OR "inter-disciplinary identit*" OR "transdisciplinary identit*" OR "trans-disciplinary identit*" OR "intergroup relational identit*" OR "team identit*" OR "dual identit*") OR ti("multi-disciplinary identit*" OR "multidisciplinary identit*" OR "cross-disciplinary identit*" OR "collaborative identit*" OR "interdisciplinary identit*" OR "interprofessional identit*" OR "inter-professional identit*" OR "interdisciplinary identit*" OR "inter-disciplinary identit*" OR "transdisciplinary identit*" OR "trans-disciplinary identit*" OR "intergroup relational identit*" OR "team identit*" OR "dual identit*")
