# Supplementary material for: Interprofessional Identity in Health and Social Care: Analysis and Synthesis of the Assumptions and Conceptions in the Literature
Source: Int J Environ Res Public Health. 2022 Nov 10;19(22):14799. doi: 10.3390/ijerph192214799 (PMC9690615; doi:10.3390/ijerph192214799)
Supplement: Supplementary file 1 [file ijerph-19-14799-s001.zip › Table S4 - List of excluded studies.pdf]

Table S4

Overview of the excluded studies with reasons (n=number of studies excluded)

Reason of exclusion		Author
Unclear description (*n*=17)	Group identity (*n*=3)	Andermo [31]
		Monrouxe [32]
		Danielson [33]
	Team identity (*n*=11)	Yank [34]
		Galeazzi [35]
		Bolíbar [36]
		Stephens [37]
		Lewin [38]
		V. Lloyd [39]
		Rodrigues [40]
		Scott [41]
		Karam [42]
		Perreault [43]
		Liebe [44]
	Collective identity (*n*=1)	Hudson [45]
	Interprofessional identity (*n*=1)	Madigosky [46]
Out of scope (*n*=5)	Interprofessional identity of researchers (*n*=1)	Timmis [47]
	Dual professional identity (*n*=1)	Sims [48]
	Teacher identity (*n*=1)	Van Lankveld [49]
	Measurement of interprofessional education (*n*=1)	Institute of Medicine [50]
	Professional identification and interprofessional collaboration (*n*=1)	Sollami [51]
Language (*n*=1)	German (*n*=1)	Swoboda [52]

Insufficient information (*n*=13)	Abstract (*n*=12)		Khalili [53]
			Sibbald [54]
			August [55]
			Ulrich [56]
			Lekka [57]
			Frasier [58]
			Bedard [59]
			Copeland [60]
			Fortin [61]
			Meyer [62]
			Hess [63]
			Sylvain [64]
Duplicate (*n*=1)	N/A		Khalili [53]
