# Supplementary material for: Interprofessional Identity in Health and Social Care: Analysis and Synthesis of the Assumptions and Conceptions in the Literature
Source: Int J Environ Res Public Health. 2022 Nov 10;19(22):14799. doi: 10.3390/ijerph192214799 (PMC9690615; doi:10.3390/ijerph192214799)
Supplement: Supplementary file 1 [file ijerph-19-14799-s001.zip › Table S5 - Data-extraction tables.pdf]

Table S5

Data-extraction form of included studies with reasons

Nr.	Citation and country	Aim	Research design and data collection	Setting and sample	Theoretical perspective and conceptual base	Antecedents	Attributes	Consequences
1	Wiles and Robison (1994) [89]<br>United Kingdom<br>*Teamwork in primary care: the views and experiences of nurses, midwives and health visitors*	To examine the various members of the nursing profession's views and experiences of teamwork and the extent to which these have been affected by recent changes to primary care.	Qualitative<br>Interviews	Practice<br>General practitioners (*n*=20), practice nurses (*n*=19), district nurses (*n*=19), health visitors (*n*=17) and midwives (*n*=17) In 20 practices in one family health services authorities	No theoretical perspective<br>No mention of interprofessional identity<br>Team identity	Intergroup leadership<br>- Control over the working situation<br>- Identification with a leader within the team<br>Interprofessional environment<br>- Frequent informal encounters with other professionals<br>- Presence in the same location as other professionals	Fluid and dynamic<br>Interprofessional values<br>- Respect<br>- Interdependence<br>- Equality<br>Interprofessional self-efficacy<br>*Interprofessional roles and responsibilities*<br>- Understanding of roles and responsibilities<br>*Teamwork and collaboration*<br>- Shared philosophy and goals	Professional wellbeing<br>- Feeling integrated in the team<br>Team effectiveness<br>- Fewer disagreements<br>- Fewer conflicts<br>- Better referrals
2	Launsø and Haahr (2007) [90]<br>Denmark<br>*Bridge building and integrative treatment of people with multiple sclerosis. Research-based evaluation of a team-building process*	To describe essential features of the preparatory phase of the research project focussing on the process of initiating and developing a team of conventual and alternative practitioners before treating people with Multiple Sclerosis.	Qualitative<br>Interviews, written materials and participatory observations of seminars	Practice<br>Conventional (*n*=5) and alternative (*n*=5) practitioners at a specialized MS hospital	Organizational learning theories<br>No mention of interprofessional identity<br>Team identity	Interprofessional environment<br>- Creation of mutually accepted norms for communication among the practitioners<br>- Development of team-based criteria of treatments<br>- An environment of trust and open communication<br>- Frequent informal encounters with other professionals<br>Breaking down barriers	Team mental model<br>- Thinking as a team<br>- Overlapping core competences<br>- Team awareness, understanding and practical approaches<br>Interprofessional values<br>- Equality<br>- Respect<br>- Interdependence<br>Awareness<br>- Not being prejudiced<br>- Being explorative and curious<br>- Not being dogmatic	Professional wellbeing<br>- Sense of team cohesiveness<br>- Improved motivation<br>- Positive emotions<br>- Sense of fulfillment<br>Health system performance<br>- Innovation and change

						- Facilitation of critical reflection upon own views and existing assumptions about own and other professions<br>- Facilitation of an open attitude and empathic understanding towards other professions<br>Interprofessional role learning<br>- Investment in building knowledge and insight about other discipline's strengths and limitations in addition to the difference and similarities between practitioners.	Openness<br>- Interprofessional openness<br>- Readiness for interprofessional collaboration<br>Interprofessional self-efficacy<br>*Interprofessional roles and responsibilities*<br>- Ability to acknowledge own limits<br>*Teamwork and collaboration*<br>- Shared philosophy and goals<br>- Ability to formulate<br>- integrative treatment plans<br>- Ability to put personal interests aside<br>- Ability to reflect on a meta-level<br>*Interprofessional communication*<br>- Ability to share knowledge and perspectives<br>- Ability to speak up<br>Interprofessional commitment	
3	Chilcutt (2009) [91]<br>United States<br>*Exploring leadership and team communication within the organizational environment of a dental practice*	To explore how leadership behaviour affects team identity, interdependence, social distance, and conflict management	Qualitative interviews (n=30)	Practice<br>Dental offices (n=10) with dentists, senior staff members and new staff members	No theoretical perspective<br>No mention of interprofessional identity<br>Team identity	Intergroup leadership<br>- A proactive leadership style<br>- Participative decision-making<br>- Use of confrontation as conflict management strategy<br>Interprofessional environment<br>- An environment of trust and open communication	Interprofessional values<br>- Interdependence<br>- Respect<br>- Equality<br>- Trust<br>Interprofessional self-efficacy<br>*Interprofessional roles and responsibilities*<br>- Ability to share decisions<br>Interprofessional commitment	Professional wellbeing<br>- Sense of team cohesiveness<br>- Improved satisfaction<br>- Feeling integrated in the team<br>- Feeling valued, empowered and

						- Regular scheduled team meetings<br>Interprofessional curricula<br>- Focus on continuing education<br>Educational strategies<br>- The use of interactive teaching methods<br>Interprofessional role learning<br>- Development of leadership, interpersonal, social, teamwork and communication skills		responsible<br>Team effectiveness<br>- Fewer conflicts<br>- Improved team communication<br>- Positive team culture
4	Mitchell et al. (2011) [92]<br>Australia<br>*When do interprofessional teams succeed? Investigating the moderating roles of team and professional identity in interprofessional effectiveness*	To explore the moderating role of team identity and professional identity threat in interprofessional team performance	Quantitative<br>Cross-sectional survey	Practice Members (*n*=218) and leaders (*n*=47) from interprofessional teams from a tertiary referral hospital	Social identity theory<br>No mention of interprofessional identity<br>Team identity	Intergroup leadership<br>- Transformational leadership<br>- Facilitation of interprofessional openness<br>Interprofessional environment<br>- Team-based rewards	Team mental model<br>- Feeling part of a superordinate group<br>- Heightened priority placed on collaboration<br>- The team as a social category<br>Interprofessional values<br>- Interdependence<br>Openness<br>- Positive beliefs towards team members<br>- Beliefs that value diversity<br>- Interprofessional openness<br>Interprofessional self-efficacy<br>*Interprofessional roles and responsibilities*<br>- Understanding of roles and responsibilities<br>*Teamwork and collaboration*<br>- Shared philosophy and goals<br>*Interprofessional*	Team effectiveness<br>- Enhanced collaborative behaviours<br>- Improved team effectiveness<br>- More innovative solutions<br>- More well-informed and comprehensive decisions

							communication<br>- Ability to share knowledge and perspectives<br>Interprofessional commitment	
5	O'Connor et al. (2015) [93]<br>United States<br>*Within-team patterns of communication and referral in multimodal treatment of chronic lower back pain patients*	To capture the features of clinician interactions within a multidisciplinary team.	Qualitative<br>Ethnography<br>Interviews, observations of interactions, and written materials	Practice<br>Clinicians surrounding CLBP patients (n=18) treated at a hospital centre of integrative medicine within the USA	No theoretical perspective<br>No mention of interprofessional identity<br>Team identity	Interprofessional environment<br>- An environment of trust and open communication<br>- Presence in the same location as other professionals<br>- Frequent informal encounters with other professionals<br>- Regular scheduled team meetings<br>Interprofessional curricula<br>- Focus on continuing education<br>Educational strategies<br>- Conjoint learning with different disciplines<br>Interprofessional role learning<br>- Investment in building knowledge and insight about other discipline's strengths and limitations in addition to the difference and similarities between practitioners	Team mental model<br>- Shared perspectives and knowledge<br>- Sense of being a team<br>Interprofessional values<br>- Equality<br>- Togetherness<br>- Interdependence<br>Interprofessional self-efficacy<br>*Interprofessional roles and responsibilities*<br>- Ability to acknowledge own limits<br>- Understanding of roles and responsibilities<br>*Teamwork and collaboration*<br>- Shared philosophy and goals<br>- Ability to gather information for treatment planning<br>- Ability to formulate integrative treatment plans<br>- Ability to share knowledge and expertise	Health system performance<br>- Higher quality of patient care<br>- Improved patient safety efforts<br>- Improved patient satisfaction
6	Caricati et al. (2015) [65]<br>Italy<br>*The role of professional and*	To analyse the effect of both professional and care unit commitments on	Quantitative<br>Cross-sectional survey	Practice<br>Physicians (n=138) and nurses (n=359) from four public	Social Identity theory<br>No mention of interprofessional identity	Intergroup leadership<br>- Facilitation of feeling part of the same team<br>- Facilitation of professionals'	Context-dependent<br>Team mental model<br>- Feeling part of the same team/superordinate category	Team effectiveness<br>- Enhanced collaborative behaviour

	team commitment in nurse-physician collaboration; a dual identity model perspective<br><br>Caricati et al. (2016) [66]<br>Italy<br>*The role of professional and team commitments in nurse-physician collaboration*	attitudes towards interprofessional collaboration between nurses and physicians		hospitals	Dual Identity<br>Team identity	motivation<br>Interprofessional role learning<br>- Learning to recognize mutual skills and abilities<br>- Learning to foster shared responsibilities	Interprofessional values<br>- Equality<br>- Respect<br>- Interdependence<br>Openness<br>- Readiness for interprofessional collaboration<br>- Positive beliefs towards team members<br>Interprofessional self-efficacy<br>*Interprofessional roles and responsibilities*<br>- Understanding of roles and responsibilities<br>- Ability to share responsibilities<br>*Teamwork and collaboration*<br>- Shared philosophy and goals<br>*Interprofessional communication*<br>- Ability to share knowledge and expertise<br>Interprofessional commitment	- Improved team effectiveness
7	Paradis et al. (2017) [94]<br>Canada<br>*Articulating the ideal: 50 years of interprofessional collaboration in Medical Education*	To determine how the notions of collaboration changed over the past 50 years in Medical Education.	Qualitative<br>Social constructionism<br>Directed content analysis	Practice<br>Collaboration-related articles (*n*=144)	Social Identity Theory<br>No mention of interprofessional identity<br>Team identity	Intergroup leadership<br>- Facilitation of social capital between team members<br>Interprofessional environment<br>- Structured debriefing procedures<br>- Regular scheduled team meetings<br>- Team-building activities that emphasize shared roles and	Context-dependent<br>Fluid and dynamic<br>Calibration<br>- Resocialisation into a more collaborative working world<br>Team mental model<br>Interprofessional values<br>- Respect<br>- Togetherness<br>- Interdependence<br>Openness<br>- Favouring of team over	Team effectiveness<br>- Enhanced collaborative behaviours<br>Health system performance<br>- Improved health outcomes

						responsibilities<br>Educational strategies<br>- Workshops comprising case studies, role plays, exercises and scenarios<br>Breaking down barriers<br>- (Early) exposure of interprofessional interactions<br>Interprofessional role learning<br>- Learning to foster shared responsibilities<br>- Development of a sense of mutual aims and common goals.<br>- Development of interpersonal, social, teamwork and communication skills<br>- Training in conflict management	individual group members<br>Interprofessional self-efficacy<br>*Interprofessional roles and responsibilities*<br>- Ability to share decisions<br>- Understanding of roles and responsibilities<br>- Ability to share leadership<br>- Ability to share responsibilities<br>*Teamwork and collaboration*<br>- Shared philosophy and goals<br>*Interprofessional communication*<br>- Ability to speak up<br>- Ability to share knowledge and perspectives	
8	Crepeau (1994) [95]<br>United States<br>*Three images of interdisciplinary team meetings*	To define and explicate the professional, constructed and ritualistic images of interdisciplinary team meetings and the influence of these images on group function	Qualitative<br>Grounded theory<br>Observations of meetings and interviews	Practice<br>Team meetings consisting of a medical director, a psychiatrist, unit director/nurse, social worker, occupational therapist<br>Interviews with medical director, unit director, and occupational therapist	No theoretical perspective<br>No mention of interprofessional identity<br>Collective identity	Interprofessional environment<br>- Rituals that bring team members together	Awareness<br>- Recognition of the value of a collaborative team approach<br>Interprofessional self-efficacy<br>*Teamwork and collaboration*<br>- Ability to formulate integrative treatment plans<br>*Interprofessional communication*<br>- Ability to share knowledge and perspectives<br>Interprofessional commitment:<br>- A sense solidarity	Professional wellbeing<br>- Sense of team cohesiveness

9	Hean and Dickinson (2005) [98]<br>United Kingdom<br>*The contact hypothesis: an exploration of its further potential in interprofessional education*	To clarify the existing use, and elaborate the future development of the contact hypothesis regarding interprofessional education	Literature review	Education<br>Medical, nursing and social work students	Social identity theory<br>No mention of interprofessional identity<br>Superordinate identity	Interprofessional curricula<br>- Institutional support<br>Breaking down barriers<br>- Challenging misperceptions, stereotypes and prejudice	Context-dependent<br>Interprofessional values<br>- Equality<br>Awareness<br>- Recognition of group similarities and differences<br>Openness<br>- Positive beliefs about team members<br>- Perceiving each other as typical members of the group<br>Interprofessional self-efficacy<br>*Teamwork and collaboration*<br>- Shared philosophy and goals<br>Interprofessional commitment<br>- A sense of belonging	Professional wellbeing<br>- Sense of team cohesiveness
10	Weller (2012) [100]<br>New Zealand<br>*Shedding new light on tribalism in health care*	To comment on the tribalism in health care through the lens of social identity	Commentary	Practice	Social identity theory<br>No mention of interprofessional identity<br>Health care professional identity	Not specified	Context-dependent<br>Team mental model<br>Interprofessional values<br>- Trust<br>Interprofessional self-efficacy<br>*Interprofessional roles and responsibilities*<br>- Ability to share responsibilities<br>*Interprofessional communication*<br>- Ability to share knowledge and perspectives<br>- Ability to speak up<br>Interprofessional commitment<br>- A sense of belonging	Team effectiveness<br>- More well-informed and comprehensiv e decisions
11	Burford (2012) [99]<br>United Kingdom<br>*Group processes*	To outline the elements of the social identity approach and	Theoretical paper	Education<br>Medical students	Social Identity Theory<br>No mention of interprofessional	Educational strategies<br>- Conjoint learning with different disciplines<br>Breaking down barriers	Context-dependent<br>Interprofessional commitment	Not specified

	in medical education: learning from social identity theory	illustrate its relevance to medical education			identity<br>Superordinate identity	- Challenging misperceptions, stereotypes and prejudice<br>Interprofessional role learning<br>- Investment in building knowledge and insight about other discipline's strengths and limitations in addition to the difference and similarities between practitioners		
12	Thistlethwaite (2016) [97]<br>Australia<br>*Interprofessional education: 50 years and counting*<br><br>Thistlethwaite et al. (2016) [96]<br>Australia<br>*Becoming interprofessional: professional identity formation in the health profession*	To comment on the state of the field at the time of publication, the impact of the article, and what we have learned since then	Commentary<br><br>Book chapter	Education<br>Medical students<br><br>Education<br>Medical students	Social identity theory, discourse and narrative, communities of practice<br>Interprofessional identity<br>Collective identity	Educational strategies<br>- Conjoint learning with different disciplines<br>- Frequent small-group works during which students talk about their profession and its roles and responsibilities<br>Breaking down barriers<br>- Challenging misperceptions, stereotypes and prejudice<br>- Guidance for students to articulate and critically reflect on their personal interprofessional values and affinities for particular ways of working, learning, or thinking<br>- Time, space and guidance for students, trainees, and other staff	Fluid and dynamic<br>- Dialogical and interactional<br>Interprofessional self-efficacy<br>*Interprofessional roles and responsibilities*<br>- Understanding of roles and responsibilities<br>*Teamwork and collaboration*<br>- Ability to acknowledge and value differences	Not specified

						for facilitating conversations about identity conflicts and ambiguous experiences within a safe and confidential environment<br>Interprofessional role learning<br>- Activities for students to develop skills in negotiation, shared decision-making, and interprofessional values-based practice		
13	Cunningham et al. (2016) [101]<br>United Kingdom<br>*GP and pharmacist inter-professional learning: a grounded theory study*	To gain general practitioners' and pharmacists' perceptions and experiences of learning together to improve care	Qualitative<br>Grounded theory<br>Focus groups and interviews	Practice<br>General practitioners (*n*=34) and pharmacists (*n*=8)	No theoretical perspective<br>No mention of interprofessional identity<br>Group identity	Interprofessional environment<br>- An environment of trust and open communication<br>- Peer support and discussing bonding events and shared experiences	Interprofessional values<br>- Trust<br>Interprofessional self-efficacy<br>*Interprofessional roles and responsibilities*<br>- Understanding of roles and responsibilities<br>*Interprofessional communication*<br>- Ability to share knowledge and perspectives<br>Interprofessional commitment<br>- A sense of belonging<br>- A sense of a unique shared history	Team effectiveness<br>- High level of understanding and trust<br>- Improved team communication<br>- Enhanced collaborative behaviours
14	King et al. (2010) [67]<br>Canada<br>*Biomechanics and evaluating the workplace: the interprofessional socialisation and*	To describe the conceptual development of the ISVS, and present findings from initial validation work	Social constructionism<br>Psychometric study	Education<br>Health professional students (*n*=124) from nursing, occupational therapy,	No theoretical perspective<br>Interprofessional identity<br>Dual identity	Intergroup leadership<br>- Nurturing of consensus in team meetings<br>- Facilitation of social capital between team members<br>- Facilitation of a learning culture	Team mental model<br>Awareness<br>- Recognition about being a team member<br>- Recognition of the benefits of implementing interprofessional practice<br>- Recognition of the	Not specified

	valuing scale: a tool for evaluating the shift toward collaborative care approaches in health care settings			physiotherapy, dietetics, speech therapists			importance of a collaborative team approach<br>- Recognition of preconceived ideas<br>- Recognition of the value of a collaborative team approach<br>Interprofessional self-efficacy<br>*Interprofessional roles and responsibilities*<br>- Understanding of roles and responsibilities<br>*Teamwork and collaboration*<br>- Ability to display collaborative skills<br>*Interprofessional communication*<br>- Ability to speak up: articulating own role and clarifying misconceptions about roles	
15	Khalili et al. (2013) [69]<br>Canada<br>*An interprofessional socialization framework for developing an interprofessional identity among health professions students*	To present a framework designed to help illuminate an IPS process, which may inform efforts by educators and curriculum developers to facilitate the development of health professions students' dual identity	Theoretical paper	Education<br>Health professions students<br><br>Education	Social identity theory<br>Interprofessional identity<br>Dual identity	Interprofessional curricula<br>- Institutional support<br>- Formal leaders and champions, governance and establish management structures<br>- a cultural shift and commitment across stakeholders<br>- Development and integration of common interprofessional competencies in professionals curricula<br>Educational strategies<br>- Conjoint learning with	Fluid and dynamic<br>Awareness<br>- Recognition of narrow uniprofessional perspective and its impact on collaboration<br>- Recognition of the value of the viewpoints and contributions of other professionals<br>Openness<br>- Interprofessional openness<br>- Readiness for interprofessional collaboration<br>Interprofessional values	Professionals experiences<br>- Improved satisfaction<br>Team effectiveness<br>- Enhanced clarity about own and other professionals<br>- Improved team communicatio n<br>Health system performance

Khalili et al. (2014) [70]<br>Canada<br>*Historical analysis of professionalism in western societies: implications for interprofessional education and collaborative practice*	To present the historical evolutions of the discourse of professionalism to assist us to develop a deeper understanding of socio-historical context within which interprofessional education is embedded within, and collaborative person centered practice	Theoretical paper			different disciplines<br>- Open interactive discussions and debates among the learners<br>- Workshops comprising case studies, role plays, exercises and scenarios<br>- Respect for learners' previous socialisation processes and perspectives when creating a climate for trusting interprofessional relationships<br>- An open and trusting environment characterised by equal status among the group members, shared goal setting, cooperating toward common goals	- Interdependence<br>- Respect<br>- Equality<br>- Trust<br>Interprofessional self-efficacy<br>*Interprofessional roles and responsibilities*<br>- Understanding of roles and responsibilities<br>*Teamwork and collaboration*<br>- Ability to formulate integrative treatment plans<br>- Shared philosophy and goals<br>*Interprofessional communication*<br>- Ability to speak up: clarify misconceptions<br>Interprofessional commitment<br>- A sense of belonging	- Improved recruitment and retention of professionals<br>- Improved patient satisfaction<br>- Improved health outcomes<br>- Improved cost-effectiveness
Khalili and Orchard (2020) [68]<br>Canada<br>*The effects of an IPS-based IPE program in interprofessional socialization and dual identity development*	To assess the effect of an interprofessional socialisation based interprofessional education program intervention on health professions students' interprofessional socialisation process and dual identity development	Mixed methods<br>Longitudinal surveys, reflection papers, and focus groups	Education<br>Students (*n*=108) from occupational therapy, food and nutrition, speech therapist, medicine, nursing, physical therapy and social work		Breaking down barriers<br>- Challenging misperceptions, stereotypes and prejudice<br>- Facilitation of critical reflection upon own views and existing assumptions about own and other professions<br>Interprofessional role learning<br>- Investment in building knowledge and insight about other discipline's strengths and limitations		

						in addition to the difference and similarities between practitioners.<br>- Students who learn to concurrently identify with their own profession and the interprofessional team		
16	Sterrett (2015) [71]<br>United States<br>*Interprofessional learning as a third space: rethinking health profession students' development and identity through the concepts of Homi Bhabha*	To seek out the ways Bhabba's views of inbetweenness enhances understanding of the student's development of an interprofessional viewpoint or identity, and to deepen the framework of an Interprofessional Community of Practice	Literature review	Education	Bhabha's concepts of hybridity and third space<br>Interprofessional identity<br>Dual identity	Educational strategies<br>- Conjoint learning with different disciplines<br>- Community of practice consisting of mutual engagement, joint enterprise, and a shared repertoire<br>Breaking down barriers<br>- Facilitation of critical reflection upon own views and existing assumptions about own and other professions	Fluid and dynamic Interprofessional commitment<br>- Hybrid culture<br>Interprofessional self-efficacy<br>*Interprofessional roles and responsibilities*<br>- Understanding of roles and responsibilities<br>Awareness<br>- Recognition about being a team member	Team effectiveness<br>- Enhanced collaborative behaviours
17	Imafuku et al. (2018) [72]<br>Japan<br>*What did first-year students experience during their interprofessional education? a qualitative analysis of e-portfolios*	To examine the undergraduate students' learning process throughout a year-long interprofessional education programme	Qualitative<br>Phenomography<br>Case study<br>Reflection papers	Education<br>Students (*n*=26) in nursing, occupational therapy, physical therapy, medicine, or dentistry	Social identity theory<br>Interprofessional identity<br>Dual identity	Educational strategies<br>- Use of e-portfolio in interprofessional modules to enhance students' reflective learning<br>Breaking down barriers<br>- (Early) exposure of interprofessional interactions	Interprofessional values<br>- Respect<br>Interprofessional self-efficacy<br>*Interprofessional roles and responsibilities*<br>- Understanding of health professionals perspective including one's own professional perspective, mutual respect, and understanding of patients' feelings and situations	Professional wellbeing<br>- Improved motivation<br>- Sense of fulfillment

							Teamwork and collaboration<br>- Understanding of the process of team development which includes leadership, mutual engagement, and consensus building<br>*Interprofessional communication*<br>- Understanding the importance of communication including active participation, active listening and intelligible explanation, through working on a interprofessional team	
18	Wang and Ho (2019) [73]<br>Taiwan<br>*Professionalism dilemmas experienced by health professions students: a cross-sectional study*	To uncover and compare professionalism dilemmas experienced by health professions students from different disciplines	Qualitative<br>Focus groups	Education<br>Students (*n*=56) in nursing, dentistry, pharmacy, medical technology, occupational therapy, and physiotherapy	Social identity theory<br>Interprofessional identity<br>Dual identity	Educational strategies<br>- Conjoint learning with different disciplines<br>Interprofessional role learning<br>- Development of leadership, interpersonal, social, teamwork and communication skills	Interprofessional commitment<br>Interprofessional self-efficacy<br>*Interprofessional roles and responsibilities*<br>- Understanding of roles and responsibilities	Not specified
19	McGuire et al. (2020) [74]<br>United States<br>*Developing an integrated interprofessional identity for collaborative practice: qualitative evaluation of an*	To examine how participation in one-credit undergraduate interprofessional education course focusing on ethical decision-making, facilitates understanding of the IPEC	Qualitative<br>Content analysis<br>Reflection papers	Education<br>Students in nursing (*n*=21), social work (*n*=14), dietetics (*n*=12), and pre-professional health students (*n*=8)	Social identity theory<br>Interprofessional identity<br>Dual identity	Interprofessional curricula<br>- Implementation of educational interventions according to the IPS-framework<br>- Development and integration of common interprofessional competencies in professionals curricula<br>Educational strategies	Fluid and dynamic<br>Interprofessional values<br>- Respect<br>- Interdependence<br>Awareness<br>- Recognition of the importance of shared interprofessional values<br>Interprofessional self-efficacy<br>*Interprofessional roles and responsibilities*	Professional wellbeing<br>Health system performance<br>- Higher quality of patient care<br>- Improved health outcomes<br>- Improved cost-

undergraduate IPE course	competencies	- Conjoint learning with different disciplines	- Ability to embrace the diversity of perspectives and development of cultural humility in working with colleagues and patients<br>- Ability to use knowledge of one’s own role and those of other professions to appropriately assess and address the healthcare needs of the patients and populations served<br>- Understanding of their role in relation to other professionals;<br>*Teamwork and collaboration*<br>- Ability to be an interdependent member of a team which integrates knowledge and perspectives from multiple sources;<br>- Understanding of the contributions of multiple disciplines in identifying and addressing complexities of ethical decision-making<br>- Ability to vision themselves in practice with others to improve health outcomes and deliver the highest quality of care<br>- Ability to work with individuals of other professions to maintain a climate of mutual respect and shared interprofessional values	effectiveness

							- Ability to apply relationship-building interprofessional values and the principles of team dynamics to perform effectively in different team roles to plan and deliver patient-centered care that is safe, timely, efficient, effective and equitable<br>*Interprofessional communication*<br>- Ability to communicate with patients, families, communities, and other health professionals in a responsive and responsible manner that supports a team approach to the maintenance of health and the treatment of disease<br>Interprofessional commitment<br>- A sense of belonging	
20	Rees et al. (2019) [75]<br>Australia<br>*Student and clinician identities: how are identities constructed in interprofessional narratives?*	To explore how final-year healthcare students and workplace-based clinicians construct their own and other's identities in interprofessional student-clinician interaction narratives.	Qualitative<br>Social constructionism<br>Focus group and interviews	Education and Practice<br>Students (*n*=38) and clinicians (*n*=23) from medicine (*n*=9), midwifery (*n*=7), nursing (*n*=11), occupational therapy (*n*=15), paramedicine (*n*=10), physiotherapy (*n*=9),	Social theories with discursive and narrative<br>Interprofessional identity<br>Dual identity	Educational strategies<br>- Focus on identity development during workplace learning experiences<br>- Frequent small-group works during which students talk about their profession and its roles and responsibilities (through use of narratives)	Context-dependent<br>Fluid and dynamic<br>Interprofessional commitment	Not specified

21	Woltenberg et al. (2019) [76]<br>United States<br>*Interprofessional identity development within a brief shadowing experience: an exploratory case study*	To examine the extent to which a brief interprofessional shadowing experience influenced interprofessional identity development	Qualitative<br>Case study<br>Reflection papers	Education<br>Students (*n*=401) from communication sciences and disorders, clinical pastoral education, dentistry, kinesiology, medicine, nursing, physician assisted studies, pharmacy, physical therapy, public health and social work	Social identity theory<br>Interprofessional identity<br>Dual identity	Interprofessional curricula<br>- Implementation of educational interventions according to the IPS-framework<br>Educational strategies<br>- Interprofessional shadowing experience with a health professional outside their own profession followed by a written reflection	Interprofessional self-efficacy<br>*Interprofessional roles and responsibilities*<br>- Understanding of roles and responsibilities<br>*Teamwork and collaboration*<br>- Ability to identify opportunities for IPC<br>Interprofessional commitment<br>- A sense of belonging	Team effectiveness<br>- Enhanced collaborative behaviours
22	Arnold et al. (2020) [77]<br>Germany<br>*Exploring early interprofessional socialization: a pilot study of student's experiences in medical history taking*	To gain insights into the experiences of cross-professional interactions and teamwork by medical and nursing students during a collective learning activity focused on joint patient history taking in interprofessional teams	Qualitative<br>Observation of recorded speed dating intervention	Education<br>Nursing (*n*=12) and medical (*n*=6) students	Social identity theory<br>Kolb's concept of experiential learning<br>Interprofessional identity<br>Dual identity	Interprofessional curricula<br>- Implementation of educational interventions according to the IPS-framework<br>- Longitudinal interprofessional education curriculum<br>Educational strategies<br>- Conjoint learning with different disciplines<br>- Collective learning activities and methods such as speed-dating	Fluid and dynamic<br>Team mental model<br>- *Sense of working side by side as a team*<br>Interprofessional values<br>- Trust<br>- Interdependence<br>- Equality<br>Awareness<br>- Recognition of the need and relevance of improved coordination<br>- Recognition about being a team member<br>Interprofessional self-efficacy<br>*Interprofessional roles and responsibilities*<br>- Understanding of roles and responsibilities	Health system performance<br>- Higher quality of patient care<br>- A more patient-centered approach

							Teamwork and collaboration<br>- Shared philosophy and goals<br>- Ability to formulate integrative treatment plans<br>*Interprofessional communication*<br>- Sharing of knowledge and perspectives<br>Interprofessional commitment<br>- A sense of belonging	
23	Tong et al. (2020) [78]<br>Australia<br>*Quality of contact counts: the development of interprofessional identity in first year students*	To measure changes in students' professional and interprofessional identities between the start and end of a faculty-wide interprofessional first year programme and identity factors influencing interprofessional identity strength at the end of the programme.	Quantitative<br>Single group pre-post-test design with surveys	Education<br>Students (*n*=658) from biomedical sciences, oral health therapy, pharmacy, midwifery, paramedicine, occupational therapy, social work, speech therapy, medicine, physiotherapy, exercise, sports and rehabilitation sciences, psychology, public health	Social identity theory<br>Interprofessional identity<br>Dual identity	Interprofessional curricula<br>- Implementation of educational interventions according to the IPS-framework<br>Educational strategies<br>- Conjoint learning with different disciplines<br>- Frequent small-group works during which students talk about their profession and its roles and responsibilities	Interprofessional commitment	Not specified
24	Trevillion and Bedford (2003) [79]<br>United Kingdom<br>*Utopianism and pragmatism in*	To determine whether the assumptive world or culture of the interprofessional curriculum is	Utopianism and Pragmatism<br>Qualitative<br>Case studies	Education<br>Students from nursing, social work and health promotors	Interprofessional identity<br>Role of change agent	Breaking down barriers<br>- Challenging misperceptions, stereotypes and prejudice<br>Interprofessional role	Calibration<br>- Loosening up or breaking with traditional boundaries<br>- Bracketing off sense of professional self and construction of a new	Not specified

	interprofessional education	influenced by the respective dominance of either a pragmatic or utopian paradigm.				learning<br>- Integrate problem solving in cases to challenge the exploration of cross-boundary issues<br>- Employ sculpting as a particular technique to enable students to link their emotional and intellectual responses together as well as to visualise patterns of interaction<br>- Develop multi-faceted understandings of specific circumstances	interprofessional self<br>- Development of a new kind of integrated interprofessional role<br>Interprofessional values<br>- Patient-centeredness<br>Interprofessional self-efficacy<br>*Teamwork and collaboration*<br>- Ability to promote partnership and collaboration<br>- Ability for problem solving cross-boundary issues<br>- Ability for sculpting<br>- Ability to work with others in difficult and conflict-laden situations<br>Interprofessional commitment<br>- A sense of being a holistic practitioner	
25	Kislov et al. (2011) [82]<br>United Kingdom<br>*Collaborations for leadership in applied health research and care: lessons from the theory of communities of practice*	To use the CoP approach as a lens to look at interprofessional and inter-organizational joint working in CLAHRCs partnerships between universities and NHS trusts	Literature review	Education and practice	Theory of communities of practice<br>No mention of interprofessional identity<br>Collaborative identity – professional identity extended with the role of boundary spanner	Intergroup leadership<br>- Presence of knowledge brokers (e.g. leaders with hybrid professional roles)<br>Interprofessional environment<br>- Use of boundary objects to facilitate knowledge transfer such as clinical pathways, assessment tools and dashboards<br>- Boundary interactions such as projects, meetings, focus groups etc	Fluid and dynamic<br>- Negotiated experiences and learning trajectory<br>Calibration<br>- Reinterpretation of experiences<br>- Stable core identity in preserving professional differences<br>- Modification of an extended identity due to widening fields of work, increased knowledge and skills, changes in attitudes and values.<br>Interprofessional self-efficacy<br>*Teamwork and collaboration*<br>- Shared philosophy and	Team effectiveness<br>- Enhanced collaborative behaviours<br>- Knowledge transfer between communities

							goals<br>Interprofessional commitment<br>- A sense of belonging	
26	Clouder et al. (2012) [103]<br>United Kingdom<br>*"Understanding where you're coming from": discovering an (inter)professional identity through becoming a peer facilitator*	To explore the range of cognitive, personal and instrumental gains for peer facilitators in the online interprofessional learning program and the extent to which participation in the project contributed to the development of a sense of professional identity and/or a superordinate interprofessional identity.	Qualitative<br>Participatory action research<br>Facilitators' application (*n*=41), interviews (*n*=37) and reflection papers (*n*=24)	Education<br>Students (*n*=41) on health and social care programs in the Faculty of Health and Life Sciences	Social Identity Theory<br>Interprofessional identity	Educational strategies<br>- Conjoint learning with different disciplines<br>Breaking down barriers<br>- Facilitation of critical reflection upon own views and existing assumptions about own and other professions	Context-dependent<br>Fluid and dynamic<br>- Intersubjective, dialogical and relational<br>Calibration<br>- Perforate boundaries<br>- Interprofessional self as a facet of professional identity<br>Openness<br>- Readiness for interprofessional collaboration<br>Interprofessional self-efficacy<br>*Interprofessional roles and responsibilities*<br>- Ability to share responsibilities<br>- Ability to share knowledge and perspectives<br>Teamwork and collaboration<br>- Ability to formulate integrative treatment plans<br>- Ability to put personal interests aside<br>- Ability to reflect on meta-level<br>Interprofessional commitment	Professional wellbeing<br>- Improved confidence<br>- Sense of freedom
27	Veerapen (2012) [80]<br>United Kingdom and Canada<br>*The impact of Uniprofessional Medical and*	To inform contemporary medical and nursing education about the impact of the socialization	Dissertation	Education<br>Undergraduate nursing (*n*=11) and medical students (*n*=11) at tertiary training	Social Identity Theory and role identity theory<br>Interprofessional identity<br>Role identity of team player	Educational strategies<br>- Cross professional mentorship<br>Breaking down barriers<br>- Early exposure of interprofessional interactions	Context-dependent<br>Calibration<br>- Sense of being part of something larger through reflection and self-awareness<br>Interprofessional values	Professional wellbeing<br>- Sense of team cohesiveness<br>Team effectiveness<br>- Enhanced

	Nursing Education on the Ability to Practice Collaboratively<br><br>Veerapen and Purkis (2014) [81]<br>United Kingdom and Canada<br>*Implications of early workplace experiences on continuing interprofessional education for physicians and nurses*	processes and the formal, informal, and hidden curriculums in professional schools, on the construction of students' interprofessional identities and interprofessional values, which in turn influence their ability to work collaboratively	Qualitative<br>Interpretive hermeneutic phenomenology<br>Interviews	hospitals in Canada and the UK		- Facilitation of an open attitude and empathic understanding towards other professions<br>Interprofessional role learning<br>- Investment in building knowledge and insight about other discipline's strengths and limitations in addition to the difference and similarities between practitioners.	- Togetherness<br>- Equality<br>- Interdependence<br>Openness<br>- Positive beliefs towards team members<br>Interprofessional self-efficacy<br>*Interprofessional roles and responsibilities*<br>- Ability to share responsibility<br>- Understanding of roles and responsibilities<br>*Teamwork and collaboration*<br>- Shared philosophy and goals<br>*Interprofessional communication*<br>- Ability to discuss patients' problems collaboratively without preparation or hesitation	collaborative behaviours<br>Health system performance<br>- Improved health outcomes
28	Oliver (2013) [83]<br>Canada<br>*Social workers as boundary spanners: reframing our professional identity for interprofessional practice*	To discuss the reframing of social workers' professional identity for interprofessional practice	Theoretical paper	Education<br>Social work students	Social Identity Theory<br>Interprofessional identity<br>Role of boundary spanner	Educational strategies<br>- Conjoint learning with different disciplines<br>Breaking down barriers<br>- Exposure of interprofessional interactions<br>Interprofessional role learning<br>- Development of leadership, interpersonal, social, teamwork and communication skills<br>- Students to learn to see themselves as	Context-dependent<br>Fluid and dynamic<br>Calibration<br>- Accessible professional identity<br>- Core element of professional identity<br>Interprofessional values<br>- Interdependence<br>- Equality<br>Openness<br>- Interprofessional openness<br>Interprofessional self-efficacy<br>*Interprofessional roles and responsibilities*<br>- Ability to acknowledge	Team effectiveness<br>- Enhanced collaborative behaviours<br>- Improved professional solidarity

						‘boundary spanners’	own limits<br>*Teamwork and collaboration*<br>- Ability to exercise non-hierarchical, facilitative leadership<br>- Ability to formulate integrative treatment plan<br>- Ability to promote partnership and collaboration<br>- Ability to acknowledge and value differences<br>*Interprofessional communication*<br>- Sharing of knowledge and perspectives<br>- Ability to break down boundaries to listen empathically and build trust<br>Interprofessional commitment	
29	Wald (2015) [102]<br>United States<br>*Professional identity (trans)formation in medical education: reflection, relationship, resilience*	To provide an overview of foundational principles and key drivers of professional identity formation	Commentary	Education	Social learning theories such as Communities of Practice<br>Interprofessional identity<br>Collaborative identity	Educational strategies<br>- Conjoint learning with different disciplines<br>Breaking down barriers<br>- Mindfulness and fostering of self-awareness<br>Interprofessional role learning<br>- Investment in building knowledge and insight about other discipline’s strengths and limitations in addition to the difference and similarities between practitioners.<br>- Students who learn to	Fluid and dynamic<br>Calibration<br>- Less bounded and more flexible identity	Professional wellbeing<br>Team effectiveness<br>- Enhanced collaborative behaviours

						concurrently identity with their own profession and the interprofessional team		
30	Langendyk et al. (2015) [85]<br>Australia<br>*Imagining alternative professional identities: reconfiguring professional boundaries between nursing students and medical students*	To explore the importance of an the pedagogical strategies used to facilitate professional identity formation for medical and nursing students	Perspective	Education<br>Medical and nursing students	Social identity theory<br>Interprofessional identity<br>Flexible professional identity	Educational strategies<br>- Conjoint learning with different disciplines<br>Breaking down barriers<br>- Challenging misperceptions, stereotypes and prejudice<br>- Mindfulness and fostering of self-awareness<br>Interprofessional role learning<br>- Development of social resilience	Fluid and dynamic<br>- Intersubjective, dialogical and relational<br>Calibration<br>Interprofessional values<br>- Patient-centeredness	Professional wellbeing
31	Stull and Blue (2016) [86]<br>United States<br>*Examining the influence of professional identity formation on the attitudes of students towards interprofessional collaboration*	To examine students' attitudes and stereotypes towards own and other healthcare professions following an interprofessional education intervention, in order to ascertain how those attitudes may affect their readiness for interprofessional learning and strength of professional	Quantitative<br>Quasi-experimental pre-post design	Education<br>Students (*n*=864) from occupational therapy, dentistry, dental hygiene, dental therapy, medicine, nursing, pharmacy, public health and veterinary medicine enrolled in an introductory interprofessiona l course	Social identity theory<br>Interprofessional identity<br>Integrated professional identity	Breaking down barriers<br>- Facilitation of critical reflection upon own views and existing assumptions about own and other professions	Calibration<br>- Element of professional identity<br>- Integration of interprofessionalism<br>- Reinterpretation of experiences and meanings<br>Interprofessional self-efficacy<br>*Teamwork and collaboration*<br>- Ability to acknowledge and value differences<br>- Ability to work with others in difficult and conflict-laden situations<br>Interprofessional commitment	Not specified

		identity						
32	Haugland et al. (2019) [84]<br>Norway<br>*Interprofessional education as a contributor to professional and interprofessional identities*	To explore, describe, and discuss the connection between interprofessional education and the development of professional and interprofessional identities	Qualitative<br>Focus groups	Education<br>Students (n=31) from nursing, radiography, occupational therapy, physiotherapy; social work, social education and biomedical laboratory sciences	Social identity theory<br>Interprofessional identity<br>Role of interprofessional	Educational strategies<br>- An open and trusting environment characterized by equal status among the group members, shared goal setting, cooperating toward common goals<br>Breaking down barriers<br>- Facilitation of critical reflection upon own views and existing assumptions about own and other professions<br>Interprofessional role learning<br>- Investment in building knowledge and insight about other discipline's strengths and limitations in addition to the difference and similarities between practitioners	Context-dependent<br>Fluid and dynamic<br>Interprofessional values<br>- Respect<br>Interprofessional self-efficacy<br>*Interprofessional roles and responsibilities*<br>- Understanding of roles and responsibilities<br>- Ability to acknowledge own limits<br>*Teamwork and collaboration*<br>- Ability to formulate integrative treatment plans<br>*Interprofessional communication*<br>- Ability to share knowledge and perspectives<br>- Ability to speak up<br>Interprofessional commitment	Not specified
33	Reinders et al. (2018) [88]<br>The Netherlands<br>*Effect of intergroup comparison of interprofessional interaction on hierarchy in mixed profession groups*	To explore the social psychological impact of task shifting between dentists and dental hygienists and to investigate the effect of an intervention that can enhance interprofessional task distribution	Dissertation	Education<br>Students (n=152) and teachers (n=48) from dentistry, dental hygiene, physiotherapy, dietetics, speech therapy, medical imaging & radiation oncology	Social identity theory<br>Interprofessional identity<br>Extended professional Identity	Educational strategies<br>- Conjoint learning with different disciplines<br>- Use reward and feedback to reinforce collective behaviours<br>- Focus on implicit next to explicit learning<br>Breaking down barriers<br>- Facilitation of critical reflection upon own views and existing assumptions about own	Context-dependent<br>Fluid and dynamic<br>Calibration<br>- Extended with interprofessional beliefs, commitment and belonging<br>Interprofessional values<br>- Respect<br>- Equality<br>- Interdependence<br>Openness<br>- Positive perceptions pertaining involvement in	Professional wellbeing<br>- Sense of team cohesiveness<br>Team effectiveness<br>- Improved team effectiveness

	and interprofessional collaboration by facilitating team formation				and other professions	goal setting, consensus planned processes, communicate plans, and joint decision making
Reinders et al. (2020) [87]<br>The Netherlands<br>*The development and psychometric evaluation of an interprofessional identity measure: Extended Professional Identity Scale (EIPIS)*	To develop and evaluate an interprofessional identity measurement instrument based on extended professional identity theory (EPIT)	Psychometric study				Interprofessional self-efficacy<br>*Interprofessional roles and responsibilities*<br>- Ability to share decisions<br>*Teamwork and collaboration*<br>- Ability to formulate integrative treatment plans<br>- Shared philosophy and goals<br>*Interprofessional communication*<br>- Ability to share knowledge and perspectives<br>Interprofessional commitment<br>- A sense of belonging
