# Supplementary material for: Interprofessional Identity in Health and Social Care: Analysis and Synthesis of the Assumptions and Conceptions in the Literature
Source: Int J Environ Res Public Health. 2022 Nov 10;19(22):14799. doi: 10.3390/ijerph192214799 (PMC9690615; doi:10.3390/ijerph192214799)
Supplement: Supplementary file 1 [file ijerph-19-14799-s001.zip › Table S6 - Code book.pdf]

Table S6

Code book used during the selection and analysis phase of the study

Antecedents	Attributes	Consequences
What directly or indirectly influences the development of an interprofessional identity?<br>- What are the proximal conditions that may facilitate the development?<br>➔ At individual level?.<br>➔ At level of education or practice?<br>- What are the methods/modulators/contextual factors in which the proximal conditions can be integrated?<br>➔ At collective level?<br>➔ At level of education or practice?	What is an interprofessional identity?<br>- What are the core elements?<br>➔ Shared values, attitudes and beliefs, knowledge?<br>- What are the characteristics?<br>➔ Fluid? Context-dependent?<br>- What is the type of identity?<br>➔ Superordinate, dual, extended, social, role?	What is the potential direct or indirect (behavioral) result of having an interprofessional identity?<br>- At group level?<br>- At individual level?<br>- Proximal or distal?

Memo's

- The ego-identity consists of multiple interrelated identities (e.g. personal, relational, social) that coexist yet become more salient according to the context.
- Data-extraction: interpretation according to the views of the articles authors and the corresponding used theory (e.g. social/professional/dual/intergroup relation identity).
  - ➔ Social identity comprises the (1) commitment to a collective, (2) sense of belonging to the group, (3) awareness of being part of the group, (4) shared feelings, beliefs and attitudes (shared mindset)
  - ➔ The professional identity is a representation of the self, achieved in stages over time, during which the characteristics, values, and norms of the respective profession are internalized, resulting in an individual thinking, acting and feeling like a professional (= more a role identity than social identity)
  - ➔ Collective identity captures the self-defined in terms of a group membership differentiated from other groups (= social identity)
  - ➔ Intergroup relational identity is defined as self-definition in terms of group membership with an emphasis on one's group's relationship with one or more other groups (=relational identity).
  - ➔ Similarity between collective identity and IRI is that they both refer to self-conception at the group level, however they differ because a collective (=superordinate identity) refers to a conception of self as a member of a singular group and implies similarity among members of this group while group members still remain distinct in the IRI.
  - ➔ In case of dual identity, group members identify strongly with both their superordinate group and subgroups and implies similarity between subgroups through the overarching superordinate identity
- *Preliminary insights on interprofessional identity*
  - ➔ *Interprofessional identity at level of an individual seems to be a role-identity*
  - ➔ *This role identity is formed through identification with an intergroup relational identity*

- ➔ *Analogous to professional (role) identity which is formed through identification with the respective professional group*

- Identity guides behavior, behavior is not part of identity but the consequence
- Some of the relevant proximal elements of identity development are congruent with the Self-Determination Theory: Autonomy, Belonging and Competence
- Link between antecedents, attributes and consequences not always clear and dependent on the
- Antecedents (e.g. interprofessional education), attributes (e.g. professional identity) and consequences (e.g. teamwork) can be related (overarching) concepts and are mostly distal

Agreements

- Antecedents are facilitators
- Articles that focus on same theoretical framework are grouped (e.g. dual identity), analyzed and reported together.
- Articles from same author are grouped, analyzed and reported together.
- Antecedents and consequences are non-exhaustive and not the purpose of this study (basis for discussion)
- Meeting after each five to ten articles
